# Tomato responses to salinity stress: From morphological traits to genetic changes

**DOI:** 10.3389/fpls.2023.1118383

**Published:** 2023-02-10

**Authors:** Mihaela Roșca, Gabriela Mihalache, Vasile Stoleru

**Affiliations:** Department of Horticultural Technologies, Faculty of Horticulture, “Ion Ionescu de la Brad” Iasi University of Life Sciences, Iasi, Romania

**Keywords:** abiotic stress, PRISMA, salt stress, screening of salinity effects, tomato, alleviation of salinity effects

## Abstract

Tomato is an essential annual crop providing human food worldwide. It is estimated that by the year 2050 more than 50% of the arable land will become saline and, in this respect, in recent years, researchers have focused their attention on studying how tomato plants behave under various saline conditions. Plenty of research papers are available regarding the effects of salinity on tomato plant growth and development, that provide information on the behavior of different cultivars under various salt concentrations, or experimental protocols analyzing various parameters. This review gives a synthetic insight of the recent scientific advances relevant into the effects of salinity on the morphological, physiological, biochemical, yield, fruit quality parameters, and on gene expression of tomato plants. Notably, the works that assessed the salinity effects on tomatoes were firstly identified in Scopus, PubMed, and Web of Science databases, followed by their sifter according to Preferred Reporting Items for Systematic Reviews and Meta-Analyses (PRISMA) guideline and with an emphasis on their results. The assessment of the selected studies pointed out that salinity is one of the factors significantly affecting tomato growth in all stages of plant development. Therefore, more research to find solutions to increase the tolerance of tomato plants to salinity stress is needed. Furthermore, the findings reported in this review are helpful to select, and apply appropriate cropping practices to sustain tomato market demand in a scenario of increasing salinity in arable lands due to soil water deficit, use of low-quality water in farming and intensive agronomic practices.

## Introduction

Tomatoes (*Solanum lycopersicum* L.) are widely consumed worldwide as fresh or processed food products (e.g. canned tomatoes, sauce, juice, ketchup, soup, etc.) (Campestrini et al., 2019; Li et al., 2021) ranking second in the top of the most consumed vegetables in the United States of America, after potatoes (Reimers and Keast, 2016). These fruits have a high content of nutrients and bioactive substances (De Sio et al., 2021; Ali et al., 2021a) that are beneficial for a healthy body, a healthy skin, and weight loss, and which may ameliorate or prevent various human chronic degenerative diseases (Ali et al., 2021a). Tomato fruits are rich in carotenoids (e.g. β-carotenoids and lycopene), ascorbic acid (vitamin C), tocopherol (vitamin E), and bioactive phenolic compounds such as quercetin, kaempferol, naringenin and lutein, caffeic, ferulic and chlorogenic acids (Dasgupta and Klein, 2014; Mihalache et al., 2020; Stoleru et al., 2020; Murariu et al., 2021). The carotenoids from tomatoes are known to display anticancer properties and to be excellent deactivators of reactive oxygen species (ROS) (e.g. for singlet oxygen (^1^O_2_) and peroxyl radical (ROO•)) (Campestrini et al., 2019; Stoleru et al., 2020). Lycopene, which is an antioxidant, might protect the cells against oxidative damage and prevent cardiovascular disease and various types of cancer (e.g. prostate, breast, lung, bladder, ovaries, colon, as well as pancreas cancer) (Dasgupta and Klein, 2014). Li et al. (2021) ascertained in their study that the consumption of tomatoes provides about 85% of the daily dose of lycopene required by the population of North America and 56–97% in five European countries.

According to FAOSTAT database, in 2020 about 251,687,023 tonnes of tomatoes were harvested from 6,163,463 hectares worldwide, with a yield average of 40.84 tonnes/ha (FAOSTAT, 2022). In 2020, the European Community reported a production of 16,657,000 tonnes, of which 9,801,000 tonnes were processed and 6,856,000 tonnes were consumed fresh. Compared to the previous year, EU production increased by almost 1%. In the last 10 years, the average annual tomato production in the EU was 16,474,000 tonnes, with the lowest value recorded in 2012 and 2013 (15,082,000 tonnes) and the highest in 2016 (17,862,000 tonnes) (European Commission, 2021).

Annually, a wide variety of factors can affect tomato yield and fruit nutritional quality (Inculet et al., 2019). Among these factors, the salt content in soil and water used in irrigation stands out. According to Shrivastava and Kumar (2015) “*worldwide 20% of total cultivated and 33% of irrigated agricultural lands* are *afflicted by high salinity*”. Furthermore, by the year 2050 more than 50% of the arable land will probably become saline soils as a consequence of weathering of native rocks, irrigation with saline water, climate change projections predicting increasing drought events forcing farmers to make use of salty water, and intensive agronomic practices. The Food and Agriculture Organization of the United Nations ascertained that every year soil salinization takes 1.5 million ha of farmland out of production and annually decreases the production potential by up to 46 million ha per year. In sum, soil salinization has been causing annual losses in agricultural productivity estimated to be US $ 31 million (FAO, 2022).

Tanji (2002) defined the salinity as “*concentration of dissolved mineral salts present in soils (soil solution) and waters*”. In small amounts, the dissolved salts are vital for the normal plant growth and development, but at high levels, they become harmful and often cause the death of plants (Nebauer et al., 2013). Sodium chloride is the most common salt detected in salty soils and waters, along with the chloride, sulfate, and carbonate salts of calcium, magnesium, and sodium (Nebauer et al., 2013; Riaz et al., 2019). Soil and water salinization generally occurs naturally, but the human factor via land clearing and inappropriate irrigation practices emphasizes this phenomenon. The soil is generally considered salt-affected when its electrical conductivity (EC) is above 4 dS·m^-1^. The soil salinity can be also increased by rainwater, which according to Riaz et al. (2019) can contain even 650 mg·kg^-1^ NaCl.

Salinity induces various deleterious effects on plants which are forced to react. Depending on the post-exposure phase, plant responses induced by salinity can be grouped into (Negrão et al., 2017; Isayenkov and Maathuis, 2019):

(I) the ion-independent response which occurs in the first hours-days after exposure and is characterized by stomatal closure and inhibition of cell expansion mainly in the shoot, and general plant growth;
(II) the ion-dependent response which takes place over days or even weeks and is characterized by the slowdown of the metabolic processes, premature senescence, and ultimately cell death.

**Figure 1:**
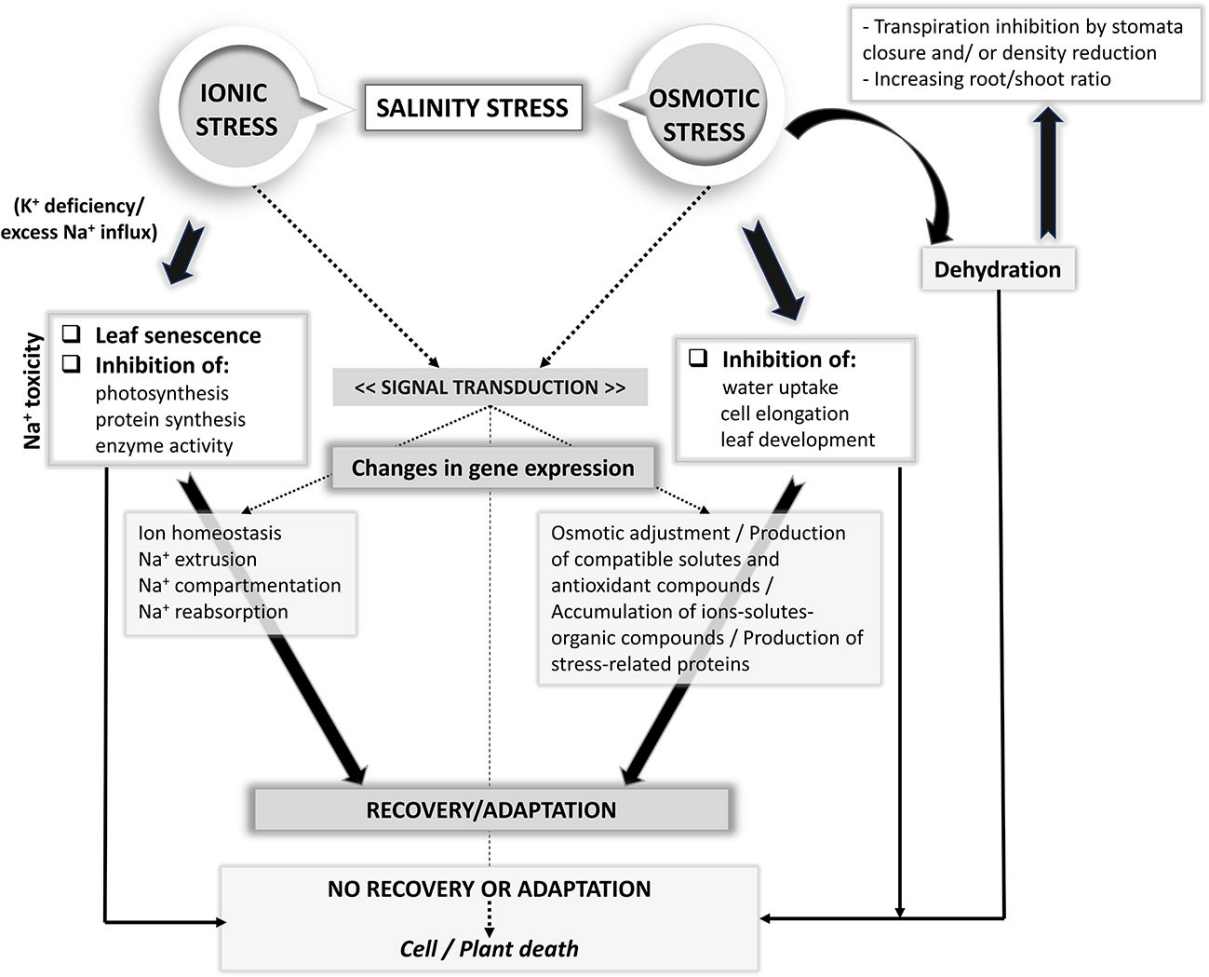
Plant adaptive responses to salt stress.

Plant adaptation to saline stress depends significantly on a multitude of physiological and molecular mechanisms which are classified into three main categories: osmotic tolerance, ion exclusion, and tissue tolerance (Munns and Tester, 2008; Roy et al., 2014; Isayenkov and Maathuis, 2019). Under salinity stress, the plants maintain their growth and development, by tolerating the water loss, preserving the leaf expansion and stomatal conductance (osmotic tolerance), avoiding the accumulation of Na^+^ ions in the shoots and leaves at toxic concentrations (by ion exclusion) and protecting the plant cells against the toxic action of Na^+^ through its removal from the cytosol and subsequent sequestration in vacuoles (tissue tolerance) (Munns and Tester, 2008; Hasegawa, 2013; Roy et al., 2014). A range of transporters and their controllers at both plasma membrane and tonoplast levels are involved in ion exclusion and tissue tolerance. The ways of plants react to salinity stress at molecular, cellular, metabolic, and physiological levels, as well as the mechanisms involved in salinity tolerance are far from being completely understood (Gupta and Huang, 2014; Maathuis, 2014). Under osmotic stress, the cell expansion in root tips and young leaves is immediately reduced and stomatal closure is induced. Plant tolerance to salt is mediated by various biochemical pathways that support water retention and/or acquisition, protection of chloroplast functions and the maintenance of ion homeostasis (Ludwiczak et al., 2021). Proline, glycine-betaine and soluble sugars are the main osmoprotectants synthesized by plants to balance the osmotic difference between the cell's surroundings and the cytosol and to protect the cell structure (Gupta and Huang, 2014; Sharma et al., 2019). According to Roy et al. (2014), the action of the tolerance mechanisms is highly dependent on the salinity level. For example, the Na^+^ exclusion is more effective in conditions of high salinity, while osmotic tolerance may be the most important tolerance mechanism at moderate salinity. In **Figure 1** the possible adaptive responses of plants to salt stress is schematically shown (Horie et al., 2012; de Oliveira et al., 2013).

Plant exposure to salinity causes negative effects on their growth and development, even leading to their death. The first visible sign of salinity stress in plants is usually stunted growth, with plant leaves often colored in bluish-green (Zahra et al., 2020). Toxicity of Na^+^ occurs with time and after a great concentration increase of these ions in the older leaves which causes their premature death (Hasegawa, 2013). Salinity induces osmotic stress, excessive uptake of sodium and chloride ions (cytotoxicity), and nutritional imbalance, impairing the plant growth and development (Zahra et al., 2020; Ludwiczak et al., 2021). Plant exposure to saline stress also causes oxidative stress due to the generation of reactive oxygen species (ROS) (Isayenkov and Maathuis, 2019). High levels of salt cause physiological dysfunctions, affect photosynthesis, respiration, starch metabolism, and nitrogen fixation, and lead to reduced crop yield (Zahra et al., 2020). Salt accumulation inside the plant tissues above the tolerance limits leads to several negative changes in plant morphology, physiology, biochemistry and crop productivity. Salinity reduces water availability for plant use and due to unfavorable osmotic pressure, the roots are unable to absorb the water (Shrivastava and Kumar, 2015). According to Hasegawa (2013), Na^+^ causes the destabilization of membranes and proteins and negatively affects the fundamental cellular and physiological processes, mainly the division and expansion, primary and secondary metabolism, and mineral nutrient homeostasis. In addition, Na^+^ competes with K^+^ uptake causing K^+^ deficiency. The adverse effects of soil salinity on plants have been proven to be caused not only by Na^+^ cations but also by Cl^−^ anions (Acosta-Motos et al., 2017). It has been reported in various studies that Cl^−^ apart from having a toxic effect on plants, it also is a beneficial element for higher plants. As a micronutrient, Cl^−^ regulates the leaf osmotic potential and turgor, stimulates growth in plants by increasing the leaf area and biomass, and improves the photosynthetic performance of plants (Colmenero-Flores et al., 2019; Franco-Navarro et al., 2019; Wu and Li, 2019). Geilfus (2018) stated that 0.2–2 mg g^–1^ fresh weight of Cl^−^ can act in stabilizing the oxygen-evolving complex of photosystem II, maintaining the electrical potential in cell membranes, regulating tonoplast H^+^-ATPase and enzyme activities. Na^+^ cations are usually more toxic than chlorine anions in plants, but Wu and Li (2019) asserted that the salinity effects observed in soybeans and avocado were mainly due to Cl^−^ toxicity. High concentrations of Cl^–^ caused nitrogen or phosphorus deficiency, interfered with photosystem II (PSII) quantum yield and photosynthetic electron transport rate, and induced necrotic lesions, resulting in the symptom of leaf-tip burning and impairment of photosynthesis and growth (Teakle and Tyerman, 2010; Wu and Li, 2019).

**Figure 2:**
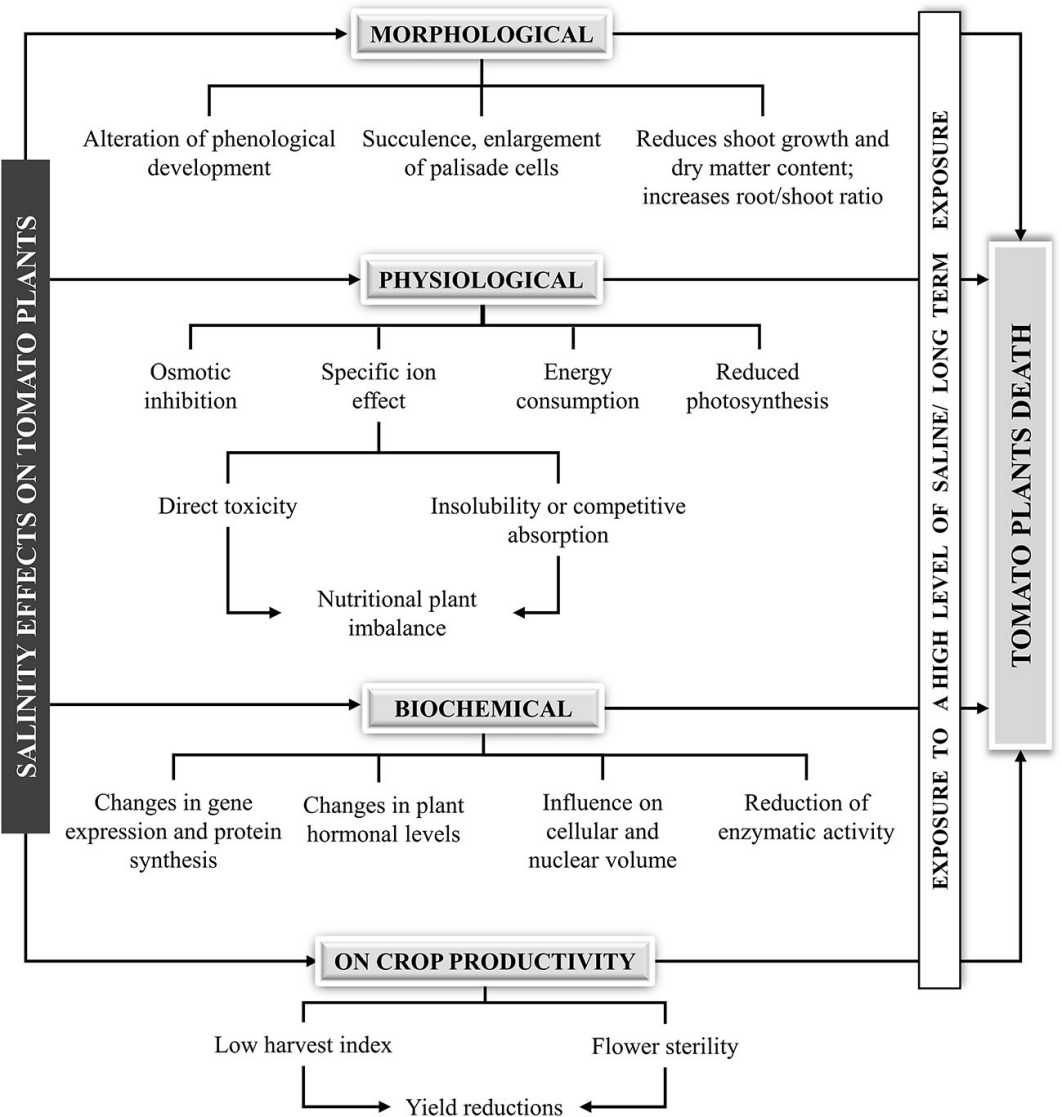
Salinity effects on tomato plants.

Due to both Na^+^ and Cl^−^ toxicity, high levels of salt can induce a large number of negative effects on tomato plants: alteration of phenological development, replacement of nutrients with sodium and chloride ions, osmotic inhibition, photosynthetic reduction, nutrient deficiencies or imbalances, changes in gene expression and protein synthesis, and negative effects on crop productivity (**Figure 2**). Salinity affects almost all aspects of plant growth including germination, vegetative growth and reproductive development. Plants are generally more sensitive to salinity during germination and early growth, and excessive accumulation of sodium in cell can rapidly lead to osmotic stress and cell death (Shrivastava and Kumar, 2015).

According to Ibrahim (2018) and Zaki and Yokoi (2016), tomato is a moderately tolerant species to salinity, and seed germination, plant growth and fruit development are just affected by high salinity levels. The response to salinity depends mainly on the tomato genotype (Zaki and Yokoi, 2016) and it has been demonstrated that salt tolerance is controlled by several gene families (Ali et al., 2021a). Studies conducted so far have highlighted that the different levels of salts in soil or in the irrigation water can induce changes in plant morphology, physiology, and biochemistry, with particular consequences on yield and fruit quality.

The knowledge of the salinity effects on tomato plants and fruits is an asset in the selection and application of the appropriate crop practices to fulfill tomato market demand. The assessment of the tomato responses to salinity stress is the main focus of this review, which was achieved through: (i) identification in Scopus, PubMed, and Web of Science databases of research works that assessed the effects induced by salinity on tomatoes, followed by (ii) their sifter according to PRISMA guideline and (iii) emphasis of the salinity effects on morphology, physiology, biochemistry, yield, fruit quality and gene expression of tomato plants induced by different levels of salts in water and soil.

## Bibliographic research and data collection

The problem of plant salinity stress has attracted the attention of many researchers who have been focusing on this topic. The main research approaches refer both to the effects of salinity on plant growth and development and to the possible strategies to increase plant tolerance to salinity. In this study, only original scientific papers which were published in the last 10 years, in peer-reviewed journals, and underlying the individual salinity effects on morphology, physiology, biochemistry, yield, fruit quality, and gene expression of tomato plants induced by different levels of Na, K and Mg salts in water and soil were included. PRISMA guideline (Page et al., 2021) was used in this review to extract from Scopus, Web of Knowledge and PubMed databases the scientific papers focused on the assessment of the effects induced by salinity on tomato plants.

**Figure 3:**
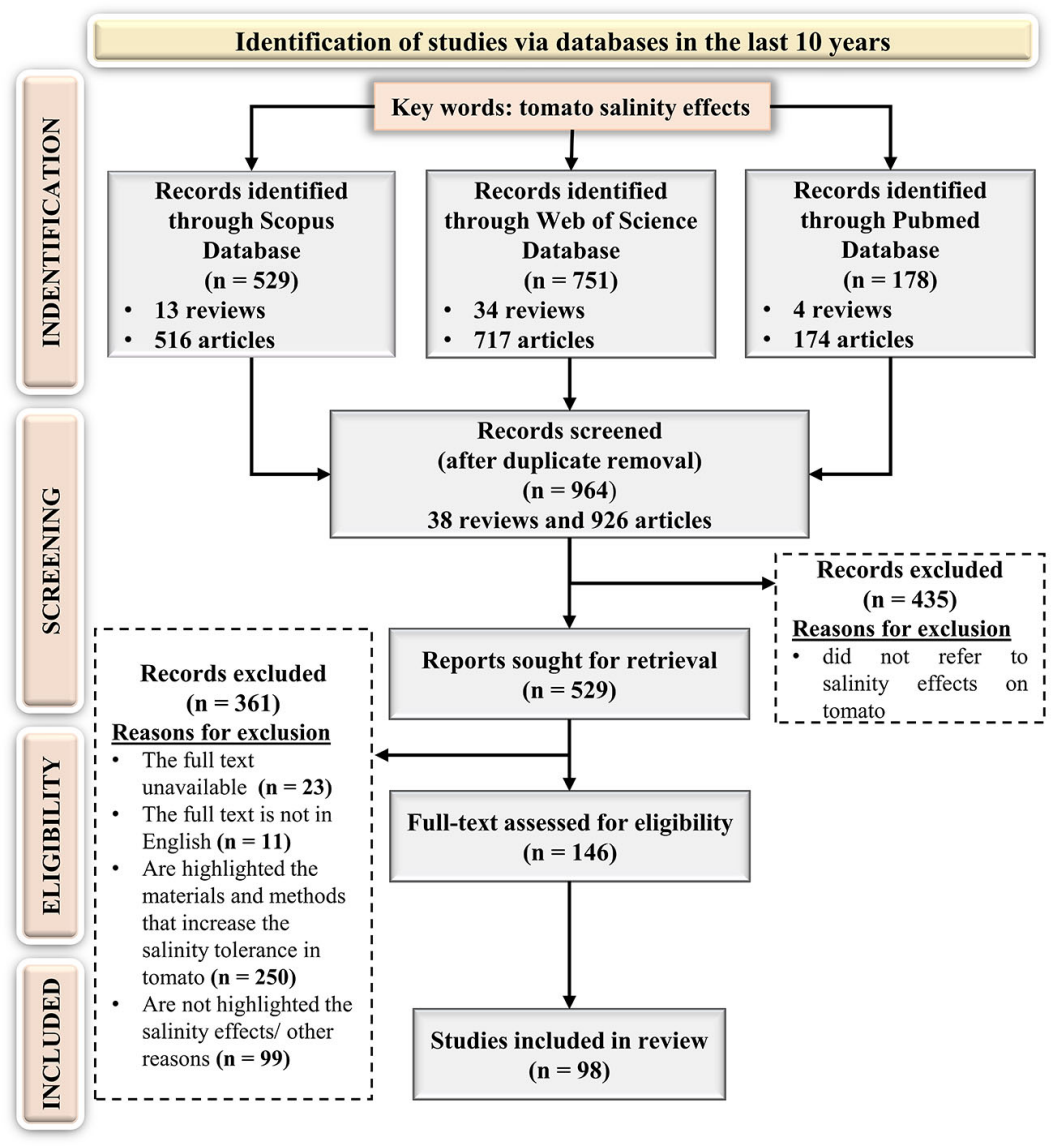
Preferred Reporting Items for Systematic Reviews and Meta-Analyses PRISMA flow diagram for the targeted systematic review.

The key expression “tomato salinity effects” was used to identify the scientific papers and the search returned 529, 751, and 178 articles in Scopus, Web of Science, and PubMed databases respectively, published in the last 10 years. According to the PRISMA flow diagram (**Figure 3**) after repetitive publications removal, 964 scientific papers were considered in the screening step. Following a careful reading of titles and abstracts, 435 articles were identified as incompatible with the search topic. Subsequently, the full texts of the left papers were downloaded and assessed to identify the works eligible with the established criteria. After an extensive screening, 11 papers in another language than English, 23 articles without full text, 250 articles focused on the methods and practices that could increase the tomato tolerance to saline stress, and 99 items for other reasons (e.g., reviews, inadequate experimental criteria data, book chapters, conference papers, are not highlighted the salinity effects, etc.) were removed.

**Figure 4:**
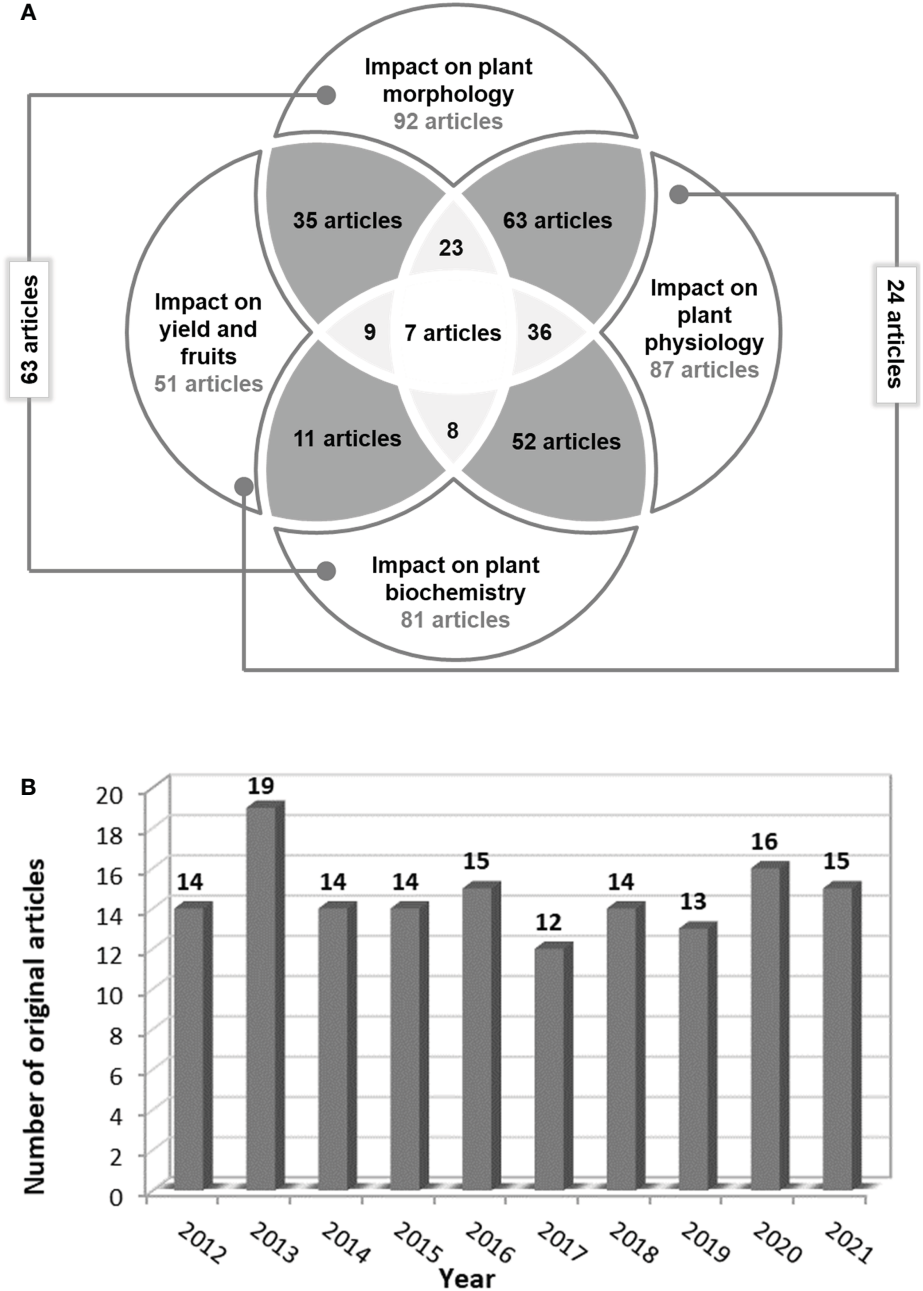
The number of relevant articles **(A)** which underline the salinity impact on tomato morphology, physiology, biochemistry or/and yield and fruits and **(B)** published annually starting from 2012.

Finally, only 146 original articles were eligible based on the inclusion criteria. The detailed analysis of these articles led to the following results (**Figure 4A**):

- 14 articles focused on salinity’s impact on seed germination;
- in 92 articles the plant/parts of the plant height, fresh/dry weight, leaf area, and/or flower/ branch number depending on salinity level in the soil or water were measured;
- in 87 articles the physiological parameters related to photosynthesis, osmosis, nutrients uptake, and water content in plant parts were evaluated;
- in 81 articles the biochemical activity of tomato plants under saline stress was assessed. The main parameters analyzed were enzymatic activity, proteins, sugars and other compound synthesis, hormonal levels, and/or molecular biology analyses.
- and only in 51 scientific papers, the impact of saline stress on yield and/or fruit quality was studied.

Out of the 146 full articles assessed for eligibility, only 98 studies were included in the reference list, following the evaluation of the information reported by the proposed objectives. In the last 10 years, at least 12 articles focusing on the impact of salinity on tomato morphology, physiology, biochemistry, and yield have been published annually in Scopus, Web of Science, and PubMed databases, respectively (**Figure 4B**).

## Morphological changes of tomato plants under salinity stress

Salinity strongly influences all the aspects of a tomato plant’s life, producing changes even in the morphological characteristics. In general, the morphology of a plant is a reflection of its environmental conditions, proving information about its metabolic function. Increases in salt content and in particular of sodium chloride in the growing environment can significantly affect the plant’s physical appearance, but also the germination traits of tomato seeds. In the study conducted by Sholi (2012), it was reported that the increase of NaCl concentration in the 1/2 MS solidified medium delayed the seed germination of all four tomato cultivars: Jenin 1, Hebron, Ramallah and Maramand. The experiments were done in Petri dishes and incubated in the light at 23^°^C. The medium with the corresponding salt concentration was solidified with 8 g L^-1^ agar. At 0 mM NaCl the time required for germination of 50 % of ‘Jenin 1’ seeds was 2.45 days, but at 100 mM NaCl the same germination rate was reached in 8.51 days. At 150 mM NaCl the germination of ‘Jenin 1’, ‘Hebron’ and ‘Maramand’ cultivar seeds were completely inhibited. Similar results were obtained by Abdel-Farid et al. (2020), who observed that a salinity level of 50, 100 and 200 mM, NaCl reduced significantly the germination rate of tomato seeds, while at 100 and 200 mM NaCl the germination of tomato seeds was completely inhibited. The authors explained that the delay in seed germination may be due to the impairment of enzyme activity by the partially osmotic or ion toxicity. González-Grande et al. (2020) found that 85 mM NaCl reduced the seed germination rate of tomato cultivar Río Grande by 6.4% compared with the control (0 mM). At 171 and 257 mM NaCl the germination was severely affected, the rate being lower than 2.8%. The experiments were done in sterile Petri dishes on filter papers. Paradoxically, at 100 mM NaCl, Tanveer et al. (2020) reported a germination rate of 80% for tomato seeds. In the study of Adilu and Gebre (2021), a delay in seed germination with salinity increase was observed, the mean germination times (days) for the four selected tomato varieties (Sirinka, Weyno, ARP D2, and Roma VF) were 10.70, 8.72, 7.31, and 6.85 days respectively at 4 dS m^-1^ and 5.79, 5.69, 4.68, 5.09 days respectively at 0 dS m^-1^. According to Adilu and Gebre (2021) a low level of NaCl induces seed dormancy while a high level inhibits seed germination. González-Grande et al. (2020); Abdel-Farid et al. (2020) and Adilu and Gebre (2021) explained that the reduction in germination rate and percentage under salt stress can be linked to a decrease in water potential gradient among seeds and their surrounding medium. Furthermore, the osmotic and toxic effects of NaCl affect the enzyme activation during seed germination and the gibberellin acid content.

Regarding the salinity effects on plant morphology, changes can appear in all stages of plant development, affecting the plant height, root/shoot ratio, leaf area, number of branches, or the number of leaves/flowers per plant. The studies focusing on the salinity effects on tomato plants showed that the intensity of plant morphology changes depends on the salt level in the growing environment. In addition, each cultivar/hybrid responds differently to saline stress. Assimakopoulou et al. (2015) assessed the responses of three cultivars (Santorini Authentic, Santorini Kaisia and Chios) and four hybrids of cherry tomato (Cherelino F_1_, Scintilla F_1_, Delicassi F_1_, and Zucchero F_1_) at 0, 75 and 150 mM NaCl in a mix of loamy soil and perlite (3:1 v/v). The results of this study showed that cultivar Chios was the most affected at 150 mM and its total plant dry weight decreased by 65.37% and the root/upper plant part ratio in terms of fresh weight from 0.09 to 0.03. The total plant dry weight of the other cherry tomato cultivars was reduced by 52.52-56.52% at the highest salinity level compared to the lowest level. Assimakopoulou et al. (2015) stated that the growth inhibition was due to the toxicity of Cl^-^ and Na^+^ ions and to the nutritional imbalance induced by salinity. Samarah et al. (2021) assessed the tomato seedling growth in response to four saline water solutions of NaCl (0, 5, 10, and 15 dS m^-1^). The seedlings at 15 dS m^-1^ had a mean length of 3.8 cm and a dry weight of 9 mg, showing a longer length and weight at 0 dS m^-1^ (16.2 cm and 45 mg/seedling, respectively).

The harmful effects of salinity on leaf area, leaf number, and leaf length also increase with the salt concentration rise, according to the studies performed by Babu et al. (2012); De Pascale et al. (2012); Hossain et al. (2012); Lovelli et al. (2012); Sánchez et al. (2012); Martínez et al. (2014); Al Hassan et al. (2015); Abouelsaad et al. (2016); Parvin et al. (2016); Chaichi et al. (2017); Rahman et al. (2018); Abdelaziz and Abdeldaym, (2019); Maeda et al. (2020). The cultivar Raf exposed at a salinity level of 5.5 dS m^-1^ had 2708 cm^2^ for the leaf area, but at 11 dS m^-1^ the leaves were smaller, and their leaf area decreased to 1815 cm^2^ (Sánchez et al., 2012). According to De Pascale et al. (2012), the saline water with an electrical conductivity of 4.4 dS m^-1^ used in tomato irrigation reduced the leaf number per plant from 82.6 at 48.9 and their leaf area with 47.55%, compared to the control. In their study, Babu et al. (2012) assessed the morphological changes induced by salinity on tomato cultivar PKM 1 based on leaf area, dry matter weight percentage, plant height and number of fruits per plant. Irrigation during 90 days with water containing NaCl at the concentrations of 0, 25, 50, 100,150, and 200 mM immediately after sowing caused negative changes in tomato plants. For example, it was found that the treatment with 200 mM NaCl reduced the plant leaf area by 43.91% and the fruit number per plant to 4 compared to 15 in the control. In addition, at this concentration, the plant height was 76.17 cm shorter compared to the control. In another study, irrigation with water having EC between 1.75 and 10.02 dS m^-1^ produced significant effects on specific leaf area, number of nodes per stem, fresh weight of roots/shoots/leaves, and length of primary roots/stem of the tomato cultivars Roma and Rio Grande (Prazeres et al., 2013). Increasing the NaCl concentration, in the irrigation water up to 3.22 dS m^-1^ led to an increase in the fresh weight of cultivar Roma leaves (by 84.7 g per plant), but at a higher NaCl concentration the leaf weight was reduced by 2.98-31.33 g. At 5.02 dS m^-1^ the leaf weight per plant was 157.80 g, with a non-significant reduction induced by salinity compared to the control whose leaf weight was 160.78 g per plant. In contrast, the fresh weight of the stems and roots decreased with the NaCl content increase in irrigation water. For cultivar Rio Grande the water EC higher than 1.75 dS m^-1^ had a positive effect on the fresh weight of roots, shoots and leaves, on specific leaf areas, number of nodes per stem and length of primary roots and stem (Prazeres et al., 2013). Several other studies have shown that the salt variation in the growing medium caused negative or positive changes in fresh biomass, plant height, root/shoot ratio, leaf areas, number of branches, and number of leaves/flowers per plant. In this respect, the results of some studies which assessed the morphological changes in tomato plants under salinity stress have been reported in **Table 1**. Reducing plant height, leaf area, leaf number, and leaf length under salt stress conditions may be an adaptive morphological strategy to limit the water loss through transpiration. However, it could also be the result of the toxicity of Na^+^ and Cl^-^ ions accumulated in cells, which slow the cell growth of young leaves (Negrão et al., 2017).

**Table 1:** Morphological changes in tomato plants under salinity stress.

Tomato cultivar	Salinity level	Plant height (cm)	Dry weight (g)	Fresh weight (g)	Root/Shoot	*Leaf area/Leaf area index*		Reference
						*unit*	value	
PKM 1	0-200 mM NaCl	161.88 ↘ 85.71	9.94 ↘ 2.79			**cm^2^**	18.24 ↘ 10.23	Babu et al. (2012)
Tainan ASVEG No. 19	0, 50, 150 mM NaCl	–	58.9 ↘ 23.0	278.4 ↘ 137	0.4 ↗ 0.5	–	–	Liu et al. (2014)
Hualien ASVEG No. 21		–	66.1 ↘ 40.4	345.7 ↘ 234.2	0.2 ↗ 0.6	–	–	
Taiwan Seed ASVEG No. 22		–	59.0 ↘ 38.0	290.3 ↘ 232.6	0.4 ↗ 0.5	–	–	
Bush Beef Steak	0, 6 dS m^-1^ NaCl	61 ↘ 60	38.5 ↘ 31.1	–	0.15 ↘ 0.14	–	–	Chaichi et al. (2017)
CF Momotaro York	1.2, 6.0 dS m^-1^ NaCl	78.4 ↗ 79 stems	99.6 ↘ 97.1		0.64 ↗ 0.85	m^2^ m^-2^	1.8 ↘ 1.7	Maeda et al. (2020)
Endeavour		128.9 ↗ 131.2 stems	109.8 ↘ 96.1		0.53 ↗ 0.83		2.2 ↘ 1.8	
Tomato	0, 100 mM NaCl	47 ↘ 35	3.53 ↘ 1.31	13.3 ↘ 3.72	0.15 ↗ 0.26	**cm^2^**	10.5 ↘ 5.2	Tanveer et al. (2020)
Roma	1.75 – 3.22 dS m^-1^	–	–	386.88 ↗ 448.03	–		–	Prazeres et al. (2013)
Rio Grande		–	–	333.80 ↗ 351.88	–	–		
Tomato	0, 2, 4, 6, 8 dS m^-1^ NaCl	88.50 ↗ 90 (0-4 dS m^-1^) 90 ↘ 75.25 (4-8 dS m^-1^)	16.48 ↘ 6.375 (stem)	–	–	**cm^2^**	187.2 ↘ 166.2	(Parvin et al., 2015)

↑, Increase in parameter value; ↓, decrease in parameter value.

The same authors interestingly focused on tissue and cellular levels to assess the morphological alterations caused by salinity in tomato plants. In this respect, Bogoutdinova et al. (2016) investigated the cell organization of the epidermis and parenchyma cortical tissues of tomato hypocotyl under different levels of NaCl *in vitro*. The size of the intercellular spaces in the cortical parenchyma as well as the average cross-sectional areas and shape of epidermal and cortical parenchyma hypocotyl cells of tomato line YaLF and cultivar Rekordsmen were significantly affected by the addition of NaCl to the culture medium. At 250 mM NaCl, the highest increase in the cell areas of tomato line YaLF was observed and the epidermal cell became angular in contours.

## Physiological changes under salinity stress

Plant physiological processes are very sensitive to all environmental changes. Variations in NaCl and other salt levels in soil or hydroponic cultivation have a strong impact on the physiology of plants. Depending on the stress duration and severity, changes that can occur in the physiological processes affect plant growth, development, and productivity. The studies done on tomatoes in the last 10 years highlighted a negative influence of salinity stress on the physiological parameters such as photosynthetic rate, transpiration, stomatal conductance, chlorophyll content and mineral uptake (Hossain et al., 2012; Lovelli et al., 2012; Giannakoula and Ilias, 2013; Maeda et al., 2020; Yang et al., 2021). For instance, Maeda et al. (2020) reported that the increase of Enshi nutrient solution EC from 1.2 to 6 dS m^-1^ caused the reduction of: photosynthetic rate by 10.2 % and 12.4 %, respectively, in tomato leaves of cultivars CF Momotaro York and Endeavour; transpiration rate and stomatal conductance by 26.9% and 23.4%, respectively, in the cultivar CF Momotaro York, and by 24.6% and 24.1%, respectively, in the cultivar Endeavour. At 6 dS m^-1^, the stomatal conductance of tomato leaves grown in silt loam soil was 0.03 mol m^-2^ s^-1^, i.e., 0.05 mol m^-2^ s^-1^ lower than in control (EC= 0 dS m^-1^ Na) (Parvin et al., 2016). Marsic et al. (2018) reported that the photosynthetic and transpiration rates as well as stomatal conductance were lower in the leaves of tomato cultivars Belle and Gardel raised in hydroponics with electrical conductivity of 6 dS m^-1^, compared to 2 dS m^-1^. The photosynthetic and transpiration rates and stomatal conductance of cultivar Belle leaves were lower by 44.1%, 52.9% and 90%, respectively, than the control, and by 40.3%, 48.6% and 91.3%, respectively compared to cultivar Gardel. According to Marsic et al. (2018), the decreased values of these parameters could be due to the stomatal closure induced by water deficit.

Like the photosynthesis rate, the chlorophyll synthesis in tomato plant leaves can be negatively affected by the exposure to high salt levels (Giannakoula and Ilias, 2013; Taheri et al., 2020). This may happen due to metabolic disorders which result in decreased chloroplast activity and photosynthesis, increased chlorophyllase enzyme activity, and respiration, followed by reduced chlorophyll contents (Taheri et al., 2020). Singh et al. (2016) found in their study that the chlorophyll content in ‘Lakshmi’ tomato leaves was reduced from 0.996 mg g^-1^ to 0.751 mg g^-1^ when the NaCl level increased from 0 to 0.5 g kg^-1^ in soilless cultivation. The same trend was observed in chlorophyll *b* synthesis, whose content decreased by 27.73% compared to the control. In another study carried out on the tomato cultivar Super Chef grown in hydroponics, the total chlorophyll content decreased by 40.93% at 120 mM NaCl compared to the control (0 mM NaCl) (Taheri et al., 2020).

The effects of salinity on photosynthesis processes in tomatowere evaluated in various studies by chlorophyll fluorescence. This type of analysis offers information on energy transfer in the photosynthetic apparatus and the related photosynthetic processes, mainly about the activity of photosystem II (PSII). PSII is a membrane protein complex whose active centers exist as dimers in the thylakoid membranes of grana stacks. It is known that PSII has the function to catalyze light-induced water oxidation in oxygenic photosynthesis and in this way light energy is converted into biologically useful chemical energy (Khorobrykh, 2019; Rantala et al., 2021). Shin et al. (2020) used chlorophyll fluorescence to assess the PSII activity in the leaves of cultivars ‘Dafnis’, ‘Maxifort’, ‘BKO’ and ‘B-blocking’ irrigated with saline water. At 400 mM (the maximum concentration of NaCl in saline water) the chlorophyll fluorescence decrease ratio (Rfd) was the parameter whose levels were most negatively affected, followed by the maximum quantum yield of PSII photochemistry (Fv/Fm). The chlorophyll fluorescence parameters, such as the coefficient of photochemical quenching of variable fluorescence based on the puddle model of PSII (qP) and coefficient of nonphotochemical quenching of variable fluorescence (qN) showed moderate negativechanges due to the salt level increase in irrigation water, whereas the quantum yield of nonregulated energy dissipation in PSII Y(NPQ) showed a significant increment at the higher salt concentration compared to control. Gong et al. (2013) reported that the values of Fv/Fm parameter and the actual quantum efficiency of photosynthetic system II (ФPSII) in cv. ‘Jinpeng No. 1’ decreased with increasing levels of salt in the hydroponic media. For the non-photochemical quenching (NPQ) parameter was noticed that an increase in salt level led to an increase in its value, the highest being recorded at 100 mM. According to Zhao et al. (2019) the qP parameter measures the openness of PSII centers and reflects the conversion efficiency of the captured light quantum into chemical energy, while qN assesses the rate constant for heat loss from PSII. Fv/Fm parameters give information about the maximum light energy conversion efficiency of PSII after adaptation to darkness and NPQ reflects the level of excess energy dissipation as heat. Using the ФPSII parameter of chlorophyll fluorescence is assessed the actual photochemical efficiency when the PSII reaction center is partly shut down under light. Thereby, as Tsai et al. (2019) and Zhao et al. (2019) stated, the changes observed in the chlorophyll fluorescence parameters under salt stress are the results of the membrane system stability disturbance (especially the damage of thylakoid membrane), the aggravation of the PSII reaction center and disturbances in PSII performance, which diminished the photosynthesis.

More results on the changes induced by saline stress on photosynthetic rate, transpiration, stomatal conductance and chlorophyll content in tomato leaves have been included in **Table 2**.

**Table 2:** Photosynthetic rate, transpiration, stomatal conductance and chlorophyll content in tomato leaves under salinity stress.

Tomato cultivar	Growth system	Salt	Salinity level (mM)	Photosynthetic rate (µmol CO_2_ m^−2^s^−1^)	Transpiration (mmol H_2_O m^−2^s^−1^)	Stomatal conductance (mol m^−2^s^−1^)	Chlorophyll (mg/g)	Reference
BINATomato-5	Hydroponic cultivation with vermiculite and half-strength Hoagland’s nutrient solution	NaCl	0, 60, 120 mM	12.2 ↘ 4.9		0.27 ↘ 0.151	–	Hossain et al. (2012)
Tomato	Hydroponic system with aerated Hoagland nutrient solution	NaCl	0, 100, 150 mM	31.3 ↘ 24.1	10.6 ↘ 5.8	0.6 ↘ 0.2	–	Lovelli et al. (2012)
Tomato	Sandy loam soil	NaCl	0, 100, 150 mg/L	25.2 ↘ 5.90	3.7 ↘ 1.39	0.5 ↘ 0.04	12.8 ↘ 7.0	Giannakoula and Ilias (2013)
Belladonna	Hydroponic system with perlite and standard nutrient solution	NaCl	0, 50 mM	25.70 ↗ 27.28	–	0.55 ↘ 0.50	–	Costan et al. (2020)
Super Chef	Hydroponic system with aerated half-strength Hoagland’s nutrient solution	NaCl	0-120 mM	–	1.373 ↘ 0.811		1.373 ↘ 0.811	Taheri et al. (2020)
Tomato	Silt loam (sand 20.84 %, silt 57.46 % and clay 21.7 %)	NaCl	0, 2, 4, 6, 8 dS m^-1^	–	–	0.08 ↘ 0.02	–	Parvin et al., (2015)
Moneymaker	Coconut coir fiber irrigated with Hoagland no. 2 solution	NaCl	0, 75 mM	8.5 ↘ 6.1	–	0.41 ↘ 0.20	–	Renau-Morata et al. (2017)
Tomato	Sandy loam soil	NaCl	0.6, 2, 4, 6 dS m^-1^	11.10 ↘ 8.86	2.65 ↘ 2.26	0.114 ↘ 0.092	39.81 ↘ 37.74	Yang et al. (2020)
CF Momotaro York	Coconut fiber and rice husks in hydroponic system with Enshi nutrient solution	NaCl	1.2, 6.0 dS m^-1^	14.7 ↘ 13.2	5.2 ↘ 3.8	0.47 ↘ 0.36	–	Maeda et al. (2020)
Endeavour				17.7 ↘ 15.5	5.7 ↘ 4.3	0.54 ↘ 0.41	–	
Belle	Vermiculite and rockwool flocks in hydroponic system with standard nutrient solution	NaCl	2, 4, 6 dS m^-1^	30.6 ↘ 17.1	25.7 ↘ 12.1	4.0 ↘ 0.4	–	Marsic et al. (2018)
Gardel				32.5 ↘ 19.4	17.6 ↘ 15.7	4.6 ↘ 0.4	–	
Ailsa Craig	Hydroponic cultivation with half-strength Hoagland’s nutrient solution	NaCl	0, 125 mM	5.97 ↘ 2.74	–	0.51 ↘ 0.25	–	Gharbi et al. (2017a)
Lakshmi	Sand	NaCl	0, 0.3, 0.5 g/kg	27 ↘ 21	–	–	0.996 ↘ 0.751 (Chl *a*) 0.256 ↘ 0.185 (Chl *b*)	Singh et al. (2016)
Tomato	Perlite-vermiculite (1:3 v/v) with half-strength modified Hoagland nutrient solution	NaCl	0, 40, 80, 160 mM	–	–	–	0.645 ↘ 0.618	Martínez et al. (2014)

↑, Increase in parameter value; ↓, decrease in parameter value.

Frequently, salinity increase can lead to a reduction in the essential minerals content such as calcium, potassium or magnesium and, consequently, to a nutritional imbalance. Calcium is one of the structural components of cell walls and membranes and serves as a second messenger in a variety of processes (Thor, 2019; Bang et al., 2021). By transduction, integration and incoming signals multiplication, the calcium links the environmental stimuli with the physiological responses of plants (Bang et al., 2021). Potassium ensures optimal plant growth, acts as an activator of dozens of important enzymes and enhances plant yield. For example, potassium plays an important role in protein synthesis, sugar transport, N (nitrogen) and C (carbon) metabolism, photosynthesis, cell osmotic pressure regulation and maintaining the balance between cations and anions in the cytoplasm (Xu et al., 2020). Magnesium in plant tissue is the central element of the tetrapyrrole ring of the chlorophyll molecule and, therefore, its deficiency leads to a chlorophyll synthesis decrease and to the impairment of normal plant growth and development. Magnesium also acts as an activator or cofactor of enzymes involved in carbohydrate metabolism (Guo et al., 2015; Bang et al., 2021). Therefore, a deficiency of these minerals in the plant tissues can cause negative effects on growth and development (Bang et al., 2021).

In tomato plants, the essential mineral uptake in soil or hydroponic cultivation can be significantly affected by saline stress (Sánchez et al., 2012; Nebauer et al., 2013; Assimakopoulou et al., 2015; Javeed et al., 2021). The results of studies presented in **Table 3** show that high salt levels in the growing culture can cause a lower uptake of calcium, potassium and sometimes of magnesium ions (Sánchez et al., 2012; Nebauer et al., 2013; Assimakopoulou et al., 2015; Parvin et al., 2016). Nebauer et al. (2013) reported in their study that regardless of the salt applied (NaCl, Na_2_SO_4_, MgCl_2_ or MgSO_4_), a level of 100 mM in soil reduced the Ca uptake by 48.75 to 71.26% in tomato cultivar Marmande RAF and by 12.28 to 38.60% in cultivar Daniela. Moreover, the amount of K in plants was lower by up to 68.05% at 100 mM MgSO_4_ in cv. Marmande RAF leaves and by up to 42.67% at 100 mM MgCl_2_ or 100 mM MgSO_4_ in cv. Daniela leaves. Decreases in the content of aforementioned minerals were also reported by Manan et al. (2016); Gharbi et al. (2017a); Rodríguez-Ortega et al. (2019) or Borbély et al. (2020). Therefore, it can be stated that salinity limits the assimilation of essential minerals in the tomato plant tissue and the physiological processes are adversely affected by these deficiencies. However, there are studies that showed that potassium, calcium and magnesium content in tomato leaves increased under salt stress (Costan et al., 2020; Javeed et al., 2021). For example, the content of calcium increased from 6.66 mg g^-1^ to 11.03 mg g^-1^ and of potassium from 36.68 mg g^-1^ to 71.51 mg g^-1^ in the fresh leaves of cultivar Rio Grande, grown in hydroponics with nutrients solution and seawater (5%, 10 % and 20%), and an EC of the growing media between 0.41 and 8.14 dS m^-1^ (Javeed et al., 2021). The high content of calcium and magnesium ions in tomato leaves under saline stress could be due to the higher uptake affinity for these ions rather than for Na^+^ or Cl^-^ (Al-Ghumaiz et al., 2017). According to Al-Ghumaiz et al. (2017), the tolerant plants under salinity stress can exclude the Na^+^ ions from their shoots or blades while maintaining high levels of K^+^.

**Table 3:** Mineral accumulation in tomato leaves under salinity stress.

Tomato cultivar	Growth system	Salinity level	Unit	Mineral content							Reference
				Na^+^	Mg^2+^	Ca^2+^	K^+^	Cl^-^	PO_4_^3-^	SO_4_^2-^	
Raf	Hydroponic system with perlite and nutrient solution	5.5 and 11 dSm^-1^ NaCl	g/kg d.w.	Leaves							Sánchez et al. (2012)
				2.71 ↗ 8.17	3.23 ↗ 3.58	24.80 ↘ 23.35	9.90 ↘ 7.98	13.49 ↗ 28.82	2.98→2.98	10.95 ↘ 8.13	
				Stem							
				2.58 ↗ 6.55	1.93 ↘ 1.81	6.96 ↘ 6.26	14.79 ↘ 12.41	15.27 ↗ 21.58	3.32 ↗ 3.42	1.83 ↘ 1.60	
Marmande RAF	Soil	25-100 mM NaCl	mmol/g d.w.	0.20 ↗ 0.158	0.33 ↘ 0.21	0.87 ↘ 0.45	0.72 ↘ 0.36	0.49 ↗ 2.22	0.19 ↘ 0.16	0.37 ↘ 0.25	Nebauer et al. (2013)
		25-100 mM Na_2_SO_4_		0.20 ↗ 2.04	0.33 ↘ 0.25	0.87 ↘ 0.42	0.72 ↘ 0.43	0.49 ↗ 0.44	0.19 ↘ 0.10	0.37 ↗ 3.48	
		25-100 mM MgCl_2_		0.20 ↘ 0.12	0.33 ↗ 1.56	0.87 ↘ 0.25	0.72 ↘ 0.31	0.49 ↗ 3.85	0.19 ↘ 0.06	0.37 ↘ 0.16	
		25-100 mM MgSO_4_		0.20 ↘ 0.10	0.33 ↗ 1.36	0.87 ↘ 0.37	0.72 ↘ 0.23	0.49 ↘ 0.44	0.19 ↘ 0.10	0.37 ↗ 2.96	
Daniela		25-100 mM NaCl		0.07 ↗ 0.65	0.17→0.17	0.57 ↘ 0.50	0.60 ↘ 0.50	0.26 ↗ 1.29	0.15 ↗ 0.20	0.29 ↘ 0.16	
		25-100 mM Na_2_SO_4_		0.07 ↗ 0.60	0.17 ↘ 0.15	0.57 ↘ 0.45	0.60 ↘ 0.42	0.26 ↗ 0.68	0.15 ↗ 0.17	0.29 ↗ 0.86	
		25-100 mM MgCl_2_		0.07 ↘ 0.05	0.17 ↗ 1.17	0.57 ↘ 0.47	0.60 ↘ 0.35	0.26 ↗ 3.44	0.15 ↘ 0.10	0.29 ↘ 0.15	
		25-100 mM MgSO_4_		0.07→0.07	0.17 ↗ 0.42	0.57 ↘ 0.35	0.60 ↘ 0.35	0.26 ↘ 0.24	0.15 ↗ 0.20	0.29 ↗ 1.01	
*Cherelino	loamy soil and perlite (3:1 v/v) and Hoagland No 2 nutrient solution	0, 75, 150 mM NaCl	g/kg d.w.	5.40 ↗ 38.10	11.00 ↘ 10.50	66.00 ↘ 53.70	25.10 ↘ 14.30	3.20 ↗ 4.50	3.30 ↘ 2.30	–	Assimakopoulou et al. (2015)
*Scintilla				5.60 ↗ 39.50	12.00 ↘ 9.80	68.10 ↘ 52.80	24.80 ↘ 18.20	2.70 ↗ 5.80	1.70 ↗ 1.90		
*Delicassi				3.70 ↗ 35.80	11.20 ↘ 10.10	59.10 ↘ 55.40	26.20 ↘ 22.10	2.00 ↗ 5.10	2.20 ↗ 2.80		
*Zucchero				5.10 ↗ 46.60	11.10 ↘ 8.90	59.20 ↘ 44.70	26.90 ↘ 14.50	2.30 ↗ 4.30	2.40 ↗ 2.70		
*Chios				13.40 ↗ 50.20	12.40 ↘ 9.40	64.30 ↘ 46.00	18.60 ↘ 7.90	2.30 ↗ 5.70	2.80 ↘ 2.30		
*Santorini Authentic				6.60 ↗ 52.10	10.40 ↘ 8.40	53.60 ↘ 43.90	17.20 ↘ 11.40	2.40 ↗ 6.70	2.60 ↘ 1.60		
*Santorini Kaisia				6.70 ↗ 48.50	12.30 ↘ 8.40	65.30 ↘ 43.20	15.80 ↘ 13.60	2.10 ↗ 6.60	1.50 ↗ 1.80		
Tomato	silt loam	0, 2, 4, 6, 8 dSm^-1^	% d.w.	0.24 ↗ 0.61	–	0.65 ↘ 0.48	1.04 ↘ 0.75	–	–	–	Parvin et al. (2016)
Rio grande	Hydroponic system with aerated Hoagland solution	0.41, 2.91, 5.96, 8.14 dS m^-1^ seawater	mg/g f.w.	11.9 ↗ 56.15	9.85 ↘ 1.35	6.66 ↗ 11.03	36.68 ↗ 71.51	8.59 ↗ 123.47	–		Javeed et al. (2021)
Belladonna	Hydroponic system with perlite and standard nutrient solution	0, 50 mmol/L	g/kg d.w.	1.51 ↗ 8.69	3.53 ↗ 4.07	16.20 ↗ 19.86	33.64 ↘ 24.78	–	3.90 ↗ 5.06	–	Costan et al. (2020)

d.w., dry weight; f.w., fresh weight. ↑, Increase in parameter value; ↓, decrease in parameter value; →, no change in parameter value.

## Salinity effects on the biochemical parameters of tomato plants and fruits

Besides affecting the morphological and physiological status, saline stress can also influence the biochemical reactions of plants. Many studies have shown that high salt concentrations cause biochemical imbalances resulting in low plant productivity (Kusvuran et al., 2016). Tomato plants, though considered moderately sensitive to saline stress, show many changes at the biochemical level such as increases or decreases in the accumulation of hormones, reactive oxygen species (ROS) or antioxidants. These changes have been mainly recorded when NaCl has been used as a salt stressor, in concentrations varying between 25 and 600 mM (**Table 4**).

**Table 4:** Salinity impact on the biochemical parameters in tomato plants and fruits.

Tomato cultivar/variety	Salinity treatment	Salt application	Plant part	Enzymatic/non-enzymatic activity	References
Perfectpeel	100 mM, 150 mM NaCl	10 days after transplantation	leaves, roots	- ABA accumulation in leaves and roots.	Lovelli et al. (2012)
PKM 1	25, 50, 100, 150 and 200 mM NaCl	immediately after sowing	leaves, fruits	- ABA, IAA accumulation in leaves; proline in fruits.	Babu et al. (2012)
*Solanum chilense*, Ailsa Craig	125 mM NaCl	23 days old	leaves	- decrease of total auxins (both cultivars) - increase of ABA (both cultivars), total jasmonates (Ailsa Craig), benzoic acid, total gibberellins, total jasmonates, cytokinins (*Solanum chilense*)	Gharbi et al. (2017a)
*Solanum chilense*,	125 mM NaCl	23 days old	leaves, roots	- increases for salicylic acid, ethylene, Spm (leaves) and Spd (roots) - decreases for Put (leaves and roots) - no impact on Spd (leaves), salicilyc acid and Spm (roots)	Gharbi et al. (2017b)
Ailsa Craig				- no significant effect on `Ailsa Craig`	
Gran Brix, Marmande Raf	100 mM NaCl	38 days after germination and was maintained for 15 days.		- increase of phytohormones: cytokinins (trans-zeatine and isopentenyl adenine), gibberellins (GA4), salicylic acid (Grand Brix); cytokinins (trans-zeatine and isopentenyl adenine), ACC, jasmonic acid, salicylic acid (Marmande), ABA - increase of the H_2_O_2_ (Marmande), LOX (Grand Brix), antioxidant enzymes, MDA -decrease of ACC, jasmonic acid, H_2_O_2_ (Grand Brix), LOX, (Marmande), catalase, ${\text{O}}_{2}^{-}$ - no effect on gibberellins (Marmande)	de la Torre-González et al. (2017b)
Micro-Tom	120 mM NaCl	20 days after cultivation	roots	- increase of MDA, carbonyl groups, glutathione reductase, glutathione peroxidase, nitric oxide. - decrease of ascorbate, glutathione, NADP-isocitrate dehydrogenase (NADP-ICDH), glucose-6-phosphate dehydrogenase (G6PDH), 6-phosphogluconate dehydrogenase (6PGDH), GSNO reductase, catalase.	Manai et al. (2014)
Cerasiforme	40, 80 and 160 mM NaCl	4 days after transplantation	leaves	- increase of SOD up to 80 mM; decrease at 160 mM - decrease of APX	Martinez et al. (2012)
Cerasiforme *S. chilense* Dun.	40, 80 and 160 mM NaCl	18 days after sowing	leaves	- no effect on total soluble proteins, MDA, caroteinoids and GR. - decrease in activity for CAT, APX (*S. lycopersicum* L. var. *cerasiforme*) - increase in activity for dehydroascorbate reductase (*S. lycopersicum* L. var. *cerasiforme*), APX and SOD (160 mM NaCl) for *S. chilense* Dun.	Martínez et al. (2014)
Puangphaka	5, 10, 25, 50 and 100 mM NaCl	starting from seed inoculation	roots	- 7 days: SOD – highest at 25 mM, lowest at 100 mM, CAT– highest at 10 mM, lowest at 50 mM and GPx – highest at 5 mM, lowest at 50 mM - 14 days: SOD – highest at 5 mM, lowest at 10 mM, CAT – highest at 5 mM, lowest at 25 mM and GPx – highest at 100 mM, lowest at 50 mM - 21 days: SOD – highest at 50 mM, lowest at 25 mM, CAT - highest at 10 mM, lowest 100 mM and GPx – highest at 10 mM, lowest at 50 mM. - the highest CAT activity at 14 days, medium at 7 days, lowest at 21 days.	Srineing et al. (2015)
			stems	- 7 days: SOD – highest at 25 mM, lowest at 100 mM, CAT - highest at 5 mM, lowest 25 mM and GPx – highest at 50 mM, lowest at 25 mM, - 14 days: SOD – highest at 5 mM, lowest at 10 mM, CAT - highest at 100 mM, lowest 5-50 mM and GPx – highest at 5 mM, lowest at 25 mM, - 21 days: SOD – highest at 50 mM, lowest at 25 mM, CAT - highest at 10 mM, lowest 100 mM and GPx – highest at 25 mM, lowest at 5 mM, - the highest CAT activity at 7 days, medium at 14 days, lowest values at 21 days.	
Cerasiforme	150, 300 and 450 mM NaCl	62 days after sowing	leaves	- no effect at 150 and 300 mM at 25 days after starting the experiment for MDA - increase for MDA at 450 mM NaCl (25 days after starting the experiment), for all the concentrations at 33 days. - decrease for total carotenoids, except 150 and 300 mM, 25 days after treatment - increase for total phenolics and flavonoids, except 150 mM, 25 days after treatment, in the case of phenolics. - decrease in TSS at 300 and 450 mM NaCl (25 and 33 days after treatment)	Al Hassan et al. (2015)
Microtom	50, 150 mM NaCl	The stage of six leaves	leaves	- increase of phenols at 150 mM	Bacha et al. (2017)
Rio grande, Savera	25, 50, 75, 100 and 125 mM NaCl	10 days after transplantation	leaves	- increase of catalase activity, peroxidase activity, total free amino acids, proline	Manan et al. (2016)
Tomato	NaCl:Na_2_SO_4_ 9:1 molar ratio	4 weeks after sowing	roots, stem, leaves	- increases of proline in the roots, stem and leaves - no change in the total sugar concentration	Wang et al. (2015)
Ciettaicale, San Marzano	300, 450 and 600 mM NaCl		leaves, roots	- increase of total antioxidant capacity at 600 mM in leaves and gradual decrease in roots for both cultivars - increase of carotenoids until 450 mM for San Marzano, gradual increase for Ciettaicale - increase of total soluble sugars in leaves of both cultivars, gradual decrease for Ciettaicale and increase until 450 mM for San Marzano in roots	Moles et al. (2016)
Tomato	25, 50, 100 and 200 mM NaCl	27 days after plantation	leaves	- increase of flavonoids, phenolics, saponin - decrease of proline, carotenoids - no effect on total antioxidant capacity	Abdel-Farid et al. (2020)
Roma, SuperMarmande	100, 200 mM NaCl	10 days after germination	leaves	- decrease of protein content at 100 mM (Roma), 100 and 200 mM (SuperMarmande). - upregulation of proteins involved in energy and carbon metabolism, photosynthesis, ROS scavenging and detoxification, stress defence and heat shock proteins, amino acid metabolism and electron transport	Manaa et al. (2013a)
Castle rock, Edkawi	50, 100, 150, 200 and 300 mM NaCl	5 days after germination	seedling	- accumulation of proteins at 50 mM, decrease at higher concentrations for Castle rock, constant accumulation for Edkawi at 100 – 200 mM, decrease at 300 mM NaCl	Khalifa (2012)
BINATomato-5	60, 120 mM NaCl	30 days after germination	leaves, roots	- accumulation of glutamate, proline, glycin, serine, alanine, protease, glutamate synthase, Fd-dependent glutamate synthase, NADP-dependent isocitrate dehydrogenase, glutamate dehydrogenase. - decrease of nitrate and nitrite reductase, soluble protein. - no change for NADH-dependent glutamate synthase	Hossain et al. (2012)
Gran brix, Marmande Raf	100 mM NaCl	38 days after germination and was maintained for 15 days.	leaves and roots	- increase in citrate synthase, malate dehydrogenase, phosphoenolpyruvate carboxylase, isocitrate dehydrogenase, citrate, malate and oxalate for Gran brix, in citrate synthase for Marmande Raf - decrease of malate dehydrogenase, phosphoenolpyruvate carboxylase, isocitrate dehydrogenase for Marmande Raf - increase of citrate (Marmande Raf) and malate for Grand brix - decrease of malate and oxalate for Marmande Raf	de la Torre-González et al. (2017a)
Ciettaicale, San Marzano	25 mM NaCl	Starting from sowing	seeds	- increase of endo-β-mannanase, β-mannosidase, catalase, total antioxidant capacity (Ciettaicale), TSS, H_2_O_2_ - decrease of endo-β-mannanase, β-mannosidase, α-galactosidase, catalase, total antioxidant capacity (San Marzano), starch	Moles et al. (2019)

ABA, abscisic acid; IAA, indole acetic acid; Spm, spermine; Spd, spermidine; Put, putrescine; ACC, aminocyclopropane-1-carboxylic acid; H_2_O_2_ - Hydrogen Peroxide; LOX, lipoxygenase; MDA, malondialdehyde; NADP, nicotinamide adenine dinucleotide phosphate; GSNO, S-Nitrosoglutathione; SOD, superoxide dismutase; APX, ascorbate peroxidase; GR, glutathione reductase; CAT, catalase; GPx, glutathione peroxidase; TSS, total soluble solids; Fd- - ferredoxin dependent; NADH, nicotinamide adenine dinucleotide.

In general, the plants respond to the salinity stress in two phases: in the first, which lasts for days or weeks, the effect of osmotic stress is predominant; in the second, of weeks to months duration, the ionic toxicity effect of leaf salt accumulation affects plant growth. In the first phase, the phytohormones play an important role in regulating plant growth. For instance, abscisic acid (ABA) under saline conditions can accumulate in tomato leaves and/or roots, as a response to the low soil water potential, causing stomatal closure, thus affecting the photosynthesis or enhancing the root growth (Babu et al., 2012; Lovelli et al., 2012; Gharbi et al., 2017a; de la Torre-González et al., 2017b). Indole acetic acid (IAA) is another hormone that is usually highly synthesized under saline stress, alleviating the negative effects of osmotic and oxidative stress, being involved in all aspects of the plant, from germination to vegetative growth and flowering. The accumulation of IAA was recorded in tomato leaves exposed to salt concentrations varying from 25 mM NaCl to 100 mM NaCl (Babu et al., 2012; de la Torre-González et al., 2017b). However, decreases or no change in the total auxins were found by Gharbi et al. (2017a), in *S. chilense* and cultivar Ailsa Craig at 125 mM NaCl or by de la Torre-González et al. (2017b) in cultivar Marmande at 100 mM NaCl. Other phytohormones studied in relation to saline stress in tomato are salicylic acid, polyamines (Put, Spd and Spm), ethylene, benzoic acid, total jasmonates, total gibberellins, cytokinins or aminocyclopropane-1-carboxylic acid (ACC, the ethylene precursor), whose content has shown very changeable responses to salinity. The content of phytohormones has been found highly dependent on the cultivar, salt concentration or plant part. For instance, the bioactive gibberellin GA4 accumulated in the cultivar Grand Brix, but not in Marmande; the total jasmonates increased in the leaves of cultivar Ailsa Craig, but remained unchanged in the roots (**Table 4**) (de la Torre-González et al., 2017b; Gharbi et al., 2017b, 2017a).

Under salinity stress, but not only, plants increased the content of ROS, causing oxidative damages. Regarding tomato, the studies have mainly focused on the activity of malondialdehyde (MDA, a lipid peroxidation marker), carbonyl groups, H_2_O_2_, ${\text{O}}_{2}^{-}$ or lipoxygenase (LOX). Their accumulation can lead to the inhibition of plant growth and development, and plant death. Increases in ROS content in tomato plants were reported at low levels of salinity (25 mM NaCl), in cultivar Ciettaicale, for hydrogen peroxide, but also at high levels of salinity (450 mM NaCl) in the variety cerasiforme for MDA (Al Hassan et al., 2015; Moles et al., 2019). The duration of exposure to salinity is an important factor in ROS accumulation, as suggested by Al Hassan et al. (2015), who recorded a significant increase in MDA content 33 days after starting the treatment but not after 25 days. Cultivar also plays a key role: the exposure of tomato cultivar Micro-Tom to NaCl (120 mM) or of Marmande and Grand Brix (100 mM NaCl) led to an increase in MDA and carbonyl groups or H_2_O_2_ and LOX contents, while at 40, 80 and 160 mM NaCl the MDA content in *S. chilense* Dun. and variety cerasiforme was not affected (Manai et al., 2014; Martínez et al., 2014; de la Torre-González et al., 2017b).

In order to prevent the negative effects of ROS, plants produce enzymatic and non-enzymatic compounds such as: ascorbic acid, phenols, ascorbate peroxidase (APX), superoxide dismutase (SOD), glutathione reductase (GR), catalase (CAT), peroxidase (POD), glutathione peroxidase (GPx), plasma glutathione peroxidase (GSHPx) etc., which play a key role in cell protection against the oxidative stress (Kusvuran et al., 2016). In tomato subjected to saline stress, the antioxidant production can vary depending on cultivar, salt concentration, plant age or part. For instance, in a study done on cerasiforme variety subjected to 40, 80 and 160 mM NaCl, the enzymatic activity of SOD increased at 40 and 80 mM NaCl, then decreased at 160 mM, while the APX activity decreased regardless of the salt concentration (Martinez et al., 2012). In another study, where tomato cultivar Micro-Tom was subjected to 120 mM NaCl, the activity/content of ascorbate, glutathione (GSH), NADP-isocitrate dehydrogenase (NADP-ICDH), glucose-6-phosphate dehydrogenase (G6PDH), 6-phosphogluconate dehydrogenase (6PGDH), S-nitrosoglutathione (GSNO) reductase and CAT decreased, while the activity of GR and GPx increased, suggesting a negative impact of the salinity stress on the redox status and NO metabolism (Manai et al., 2014). Interesting findings were made by Srineing et al. (2015), in a study *in vitro* on the cultivar Puangphaka treated with NaCl at concentrations ranging between 5 – 100 mM. The authors analyzed the activity of SOD, CAT and GPx (roots and stem) at different time intervals: 7, 14, 21 days after incubation. The results showed differences in enzyme activity depending on plant age and part (roots or stems) (**Table 4**). The influence of the salt and the exposure time on total carotenoids, total phenolics, total flavonoids and TSS was also analyzed by Al Hassan et al. (2015) in cerasiforme variety exposed to 150, 300 and 450 mM NaCl. The results showed that regardless of the time of treatment (25 or 33 days) the content of total carotenoids significantly decreased at all the concentrations, except for 150 and 300 mM, 25 days after treatment, while the content of the total phenolics and flavonoids significantly increased at all the salt concentrations, except for 150 mM, 25 days after treatment, in the case of phenolics. In another study, where the tomato plants of cultivar Microtom were exposed shorter to NaCl stress (14 days) the phenols increased to 150 mM NaCl (Bacha et al., 2017). Changes in the antioxidant activity were also reported by Martínez et al. (2014); Manan et al. (2016) and de la Torre-González et al. (2017b), included in **Table 4**.

Salinity stress is known to produce a C shortage in plants, stimulating the synthesis of C-rich compounds such as trehalose, mannitol, sorbitol or proline, involved in the osmotic adjustment mechanism to stressful conditions. Moreover, the N status is affected because of the influence on ${\text{NO}}_{3}^{-}$ and ${\text{NO}}_{4}^{+}$ uptake.

Hossain et al. (2012) and Manai et al. (2014) reported that the activity of enzymes involved in the N absorption was affected by saline stress: a decrease was recorded for nitrate and nitrite reductase or nitric oxide (NO), suggesting a negative impact on the NO metabolism under salinity stress, while an increase was recorded for protease, glutamate synthase and Fd-dependent glutamate synthase, NADP-dependent isocitrate dehydrogenase, and glutamate dehydrogenase. No change was observed for NADH-dependent glutamate synthase. Most of the studies carried out on different tomato cultivars, varieties or genotypes (e.g. BINATomato-5, PKM1, Cerasiforme, Rio grande, Savera, Ciettaicale or San Marzano) reported increases in the proline, glycine betaine, serine, alanine, or total soluble sugars contents under different NaCl concentrations, as a result of osmotic adjustments (Babu et al., 2012; Hossain et al., 2012; Al Hassan et al., 2015; Manan et al., 2016; Moles et al., 2016). Increases in the proline content in the roots, stems and leaves of tomato plants, but not of the total soluble sugars, were also recorded in the case of combined salt stress, consisting of NaCl:Na_2_SO_4_ in a molar ratio of 9:1 (Wang et al., 2015). By contrast, a decrease in the proline content was reported by Abdel-Farid et al. (2020), in a pot experiment, where tomato plants were treated with 25, 50, 100, 200 mM NaCl. The decrease was explained by taking into consideration the replacement of the proline by another osmoprotectant under saline conditions.

The salinity stress can also affect the protein content of plants. A study performed on two tomato cultivars (Castle rock and Edkawi) with different tolerance to salinity showed an accumulation of proteins (the large chloroplast subunit (RbcL), structural maintenance of chromosomes (SMC) protein, a protein from the plasma membrane, and transcription factors) at 50 mM NaCl in both cultivars, a gradual decrease at higher salt concentration for Castle rock and an approximately constant accumulation for Edkawi at 100, 150, 200 mM NaCl, followed by a decrease to 300 mM NaCl. According to the authors, the accumulation of RbcL at 50 mM NaCl in the cultivar Castle rock might be the result of Rubisco degradation under saline stress, as this cultivar is more sensitive to salinity. The better tolerance to salt stress of cultivar Edkawi is demonstrated by better retention of Rubisco content, chromosome segregation and up-regulation of ion pump proteins (Khalifa, 2012). In another study carried out on the cultivar BINATomato-5 the soluble protein content decreased by 25.64% at 60 mM NaCl and by 42.75% at 120 mM NaCl (Hossain et al., 2012). A decrease in protein content was also observed by Manaa et al. (2013a) in the leaves of two tomato cultivars (Roma – salt tolerant, SuperMarmande – salt sensitive), at 100 and 200 mM NaCl. The same author conducted leaf proteomic analysis, identifying 26 proteins involved in energy and carbon metabolism, photosynthesis, ROS scavenging and detoxification, stress defense and heat shock proteins, amino acid metabolism and electron transport. The majority of the proteins identified were upregulated as a consequence of saline stress. Variations in protein abundance were also reported in the fruits of two tomato cultivars (Cervil and Levovil), which were correlated to the salt treatments and the fruit ripening stage. Most of the proteins identified were associated with carbon and energy metabolism, salt stress, oxidative stress, and the ripening process (Manaa et al., 2013b). In general, the content of soluble proteins represents an indicator of plant physiological status under stress, having an important role in osmotic adjustments, and providing storage for different forms of nitrogen. Depending on the cultivar, the soluble proteins can decrease as a result of protein synthesis inhibition and/or protein hydrolysis or can increase through the production of new stress-related proteins (Ahmad et al., 2016).

Salinity stress can also have no impact on the protein content, as recorded by Martínez et al. (2014), in a study done on *S. chilense* Dun. and variety cerasiforme at 40, 80, or 160 mM NaCl.

Salinity can also affect the carboxylate metabolism and organic acid production, depending on the cultivar as demonstrated by (de la Torre-González et al., 2017a) (**Table 4**). High activity of the enzymes involved in the carboxylate metabolism enhances tomato resistance to salinity due to the activation of osmotic adjustments mechanism of response which helps the plant to adapt to stressful conditions. Also, high organic acid concentrations are necessary for enhancing the plant’s tolerance to salinity, taking into account their important role in different biochemical pathways, such as energy production or amino-acid biosynthesis. In addition, Moles et al. (2019) showed that NaCl can influence the activity of the cell wall enzymes (endo-β-mannanase, β-mannosidase, α-galactosidase) involved in seed germination. Under 25 mM NaCl, the concentration of endo-β-mannanase and β-mannosidase increased in cultivar Ciettaicale, and decreased in cultivar San Marzano affecting the seed germination. Reyes-Pérez et al. (2019) stated that acid and alkali phosphatase, trypsin, lipase, β-galactosidase, and esterase can be used as biomarkers for NaCl-stress tolerance in tomato.

## Salinity effects on tomato gene expression

In general, salinity stress, like other abiotic stresses, determines changes in the gene expression of plants. The knowledge of the gene expression as a result of salt stress is still limited, but mostly refers to changes in transcription factors (Devkar et al., 2020).

Tomato research regarding the effect of salinity on gene expression has been carried out on different cultivars and focused mostly on the effect of NaCl applied at the concentration range between 50 and 500 mM (**Table 5**). The results suggested changes in the expressions of genes involved in cell wall construction, biosynthesis of volatiles and secondary metabolites, protein synthesis, transport activity, etc. for the plants subjected to salinity stress.

**Table 5:** Salinity stress-related genes in tomato plants.

Tomato cultivar/accessions	Salinity level	Stress-related genes	References
Micro-Tom	100–400 mM NaCl	*4CL3, PAL6, CHI1, CHI2, HQT, XTH4, XTH20, XTH16, EXPA4, EXPA5, EXPA18, FLA 2, FLA10, FLA11, TPS, FPS, LEA, LOX, HSF30*	Hoffmann et al. (2021)
Yanfen 210	10%, 20%, 30%Seawater	*SlGA20OX1, SlMYB13, SlCI-2, SlHYD, SlPCC27-04, SlMYB48, SlAPRR5, SlMFS*	Mu et al. (2021)
Ailsa Craig	150 mM NaCl	*SlSOS2, P5CS, SlDREB2*	Coyne et al. (2019)
Tomato	0, 50, 75 mM	*CS, AH, PDH, PAP, ALDH, DGD, LAT, DGK, FAD, LCS, ACOX, PHS, AOS, FPS, MK, GPS*	Zhang et al. (2018)
New Yorker	0.2 M NaCl/0.02 M CaCl_2_	*NCED1, TAS14*	Pye et al. (2018)
Manitoba	100 mM NaCl	*NRT1.1, NRT1.2, AMT1.1, AMT1.2, Gs1*	Abouelsaad et al. (2016)
*Solanum chilense* LA 1938, LA 1959; *S. chmielewskii* LA 1325, LA 2695; *S. corneliomuelleri* GI 568, PI 126443; *S. galapagense* LA 0532, LA 0317; *S. habrochaites* G156, LA 2167; *S. habrochaites glabratum* LA 2860, PI 126449; *S. lycopersicum* Abigail F1, LA 3320, LA 2711 and Arbasson F1; *S. neorickii* LA 2194; *S. pennellii* LA 1340, LA 1522; *S. pennellii puberulum* LA 1302; *S. peruvianum* LA 2548; *S. pimpinellifolium* OT 2209, LA 1245	100mM NaCl	*P5CS, NHX1, NHX3, HKT1;1, HKT1;2, SOS1*	Almeida et al. (2014b)
Arbasson	0, 5, 75 mM NaCl	*HKT1;2*	Almeida et al. (2014a)
Ailsa Craig	100, 200, 300, 400, 500 mM NaCl	*SlERF5*	Pan et al. (2012)
Rio Fuego	100 mM NaCl	*SlGSTU23*, *SlGSTU26, SlGSTL3, SlGSTT2, SlDHAR5, SlGSTZ2*	Csiszár et al. (2014)
San Miguel, Perfect peel HF1, Mouna HF1	150 mM NaCl	*WRKY (8, 31, 39), ERF (9, 16, 80), LeNHX (1, 3, 4), HKT (Class 1)*	Gharsallah et al. (2016)
Mircrotom	0, 10, 20, 25, 30 mM Na^+^	*LeHAK5*	Bacha et al. (2015)
Microtom	250 mM NaCl	*SlARF1, SlARF4, SlARF8A, SlARF19, SlARF24*	Bouzroud et al. (2018)

4CL3, 4-coumarate-CoA ligase; PAL6, phenylalanine ammonia lyase; CHI1, CHI2, chalcone isomerase; HQT - hydroxycinnamoyl-CoA quinate transferase; XTH4, XTH20, XTH16, xyloglucan endo-transglucosylase or hydrolase; EXPA, expansins; FLA, fasciclin-like arabinogalactan proteins; TPS, terpene synthase; FPS, farnesyl diphosphate synthase; LEA, late embryogenesis abundant proteins; LOX, lipoxygenase genes; HSF30, heat shock transcription factor; SlGA20OX1, gibberellin 20-oxidase1 gene; SlMYB, transcription factors of the MYB family; SlCI-2, proteinase inhibitor; SlHYD, SlMFS, genes related to membrane; SlPCC27-04, desiccation-related protein; SlAPRR5, response regulator; SlSOS2, Solanum lycopersicum Salt-Overly-Sensitive 2; P5CS, pyrroline 5-carboxylate synthase, SlDREB2, Solanum lycopersicum Dehydration Responsive Element Binding 2; CS, citrate synthase; AH, aconitate hydratase; PDH, pyruvate dehydrogenase; PAP, phosphatidate phosphatase, ALDH, phosphatidate phosphatase, DGD, digalactosyldiacylglycerol synthase, LAT, lysophospholipid acyltransferase, DGK, diacylglycerol kinase, FAD, fatty acid desaturase, LCS, long chain acyl-CoA synthetase, ACOX, peroxisomal acyl-CoA oxidase, PHS, beta-phellandrene synthase, AOS, allene oxide synthase, MK, mevalonate kinase, GPS, geranyl pyrophosphate synthase; NCED1, 9-cis-epoxycarotenoid dioxygenase; TAS14, tomato dehydrin gene; NRT, nitrate transporters; AMT, ammonium transporters; Gs1, glutamine synthetase; NHX, Na^+/^H ^+^ Antiporters; HKT1;1; HKT1;2, sodium transporter; SOS1, Salt-Overly-Sensitive 1; SlERF5, ethylene response factors; SlGSTU23, SlGSTU26, SlGSTL3, SlGSTT2, SlDHAR5, SlGSTZ2, Solanum lycopersicum glutathione-S-transferase genes; ERF , ethylene responsive factor HKT, Histidine Kinase Transporter; LeHAK5, potassium transporter; SlARF, Solanum lycopersicum auxin response factor.

In a study with the cultivar Micro-Tom subjected to NaCl at 100, 200 and 400 mM, the genes responsible for the phenylpropanoid pathway (*4CL3* = 4-coumarate-CoA ligase, *PAL6* = phenylalanine ammonia lyase, *CHI1* and *CHI2* = chalcone isomerase, *HQT* = hydroxycinnamoyl-CoA quinate transferase), xyloglucan endo-transglucosylase or hydrolase (*XTH4*, *XTH20*, *XTH16*) activities, or enzymatic response to reactive oxygen species (*ROS*, *SOD* genes), were up-regulated in the top younger leaflets as compared to the older ones situated at the bottom of tomato plants, indicating an increase in the lignification process and flavonoid synthesis, a strengthening in the mechanical cell wall properties and an intensification in SOD production, an enzyme involved in the response to ROS as a result of the salinity stress. Furthermore, in the top leaflets of stressed plants, the expression of expansins (*EXPA4*, *EXPA5*, *EXPA18*), genes involved in cell wall reshaping, fasciclin-like arabinogalactan proteins (*FLA 2, FLA10, FLA11*) involved in keeping the plasma membrane and cell wall in close contact, and volatile organic compounds’ synthesis (*TPS*, *FPS*) were down-regulated, suggesting an increase in the salt sensitivity, as plant growth was stopped, as well as the production of terpene synthase (*TPS*) or farnesyl pyrophosphate synthase (*FPS*). Changes in the gene expression were also recorded in the bottom leaflets, with the LEA and LOX genes up-regulated, indicating an accumulation in late embryogenesis abundant (LEA) proteins responsible for membrane maintenance and ion-sequestering properties, as well as in lipoxygenases, markers for cell membrane damage.

Other up-regulated genes in the salt-stressed tomato plants were those coding for heat shock transcription factor *HSF30* (Hoffmann et al., 2021). In another experiment, in which tomato cultivar Yanfen 210 was treated with seawater at different concentrations (10%, 20% and 30%), a significant differential change was recorded in the expression of 509 genes, 40.67% of which were up-regulated, while 59.33% down-regulated. The highlighted genes were responsible for biological processes (i.e. metabolic process, cellular process or single organism process), cellular components (i.e. cell, cell part, membrane, organelle, etc.) or molecular functions (i.e. catalytic activity, binding, transporter activity, etc.). Notably, the *SlGA20OX1*gene expression was down-regulated, thus affecting the production of gibberellin and plant growth. Down-regulations were also observed for *SlMYB13*, part of MYB family transcription factors involved in biological and developmental processes, cell morphology, biological stress response, primary and secondary metabolism adjustment, *SlCI-2* gene involved in the inhibition of proteinase activity or *SlHYD* gene responsible for the activity of cell membrane. On the other hand, over-expressions were observed for *SlPCC27-04* gene coding for plant desiccation-related proteins, *SlMYB48* gene responsible for ABA signaling, *SlAPRR5* gene known to control the time of the flowering process, the circadian rhythms or the photomorphogenesis, or *SlMFS* gene involved in the membrane activity (Mu et al., 2021). Zhang et al. (2018), investigating the effect of NaCl on the volatile compound emission of tomato plants, found the expression of 18 genes down-regulated, thus affecting the biosynthesis of isopentenyl diphosphate isomerase, geranyl pyrophosphate synthase, sesquiterpene synthase, β-phellandrene synthase, terpene synthase 1, 28, 38 or farnesyl pyrophosphate synthase 1. Out of a total 7210 differentially expressed after NaCl exposure, of which 1208 were over-expressed and 6200 were down-expressed, other 3454 genes were related to plant-pathogen interaction, RNA-transport or hormone signal transduction. Changes in the expression of hormone-related genes were also recorded by Pye et al. (2018) in the roots of the cultivar New Yorker. The treatment with NaCl and CaCl_2_ led to an increased expression of two ABA-related genes: *NCED* and *TAS14*.

An interesting finding was made by Coyne et al. (2019), who observed a correlation between the expression of some genes and the circadian rhythms. The gene coding for sodium or hydrogen antiporter and an enzyme for proline synthesis, *SlSOS2* and *P5CS*, were expressed only in the morning, while *SlDREB2* encoding a transcription factor responsible for the response of tomatoes to salinity was expressed only in the evening. Due to this behavior, tomato, but also other species, might be able to keep the balance of the endogenous systems to circadian rhythms. Almeida et al. (2014b) also reported an overexpression of *P5CS* gene which led to an accumulation of proline and Na^+^ in the leaves of five weeks old tomato plants, but not in the roots. The same authors observed a higher expression of *NHX1* and *NHX3* genes correlated with a lower Na^+^ accumulation in leaves, and a higher Na^+^ accumulation in roots; the expression of *HKT1;2* gene in the roots was positively correlated with the amount of Na^+^ in leaves and stems, but not in the roots, where other genes were responsible for the accumulation of Na^+^ (*HKT1;1*). Changes in the expression of *HKT1;2* gene due to salinity stress was also recorded in the cultivar Arbasson where an increase in the gene expression in stems and roots was recorded along with increased salinity stress. In leaves, the accumulation of Na^+^ was correlated with a low expression of *HKT1;2* genes (Almeida et al., 2014a). The role of *HKT1;1* and *HKT1;2* in the ion homeostasis in tomato leaves and stems was also confirmed by Asins et al. (2013). Jaime-Pérez et al. (2017) demonstrated in transgenic tomato plants the importance of *HKT1*;*2* gene in Na^+^ homeostasis and salinity tolerance. The same genes (*HKT1;1* and *HKT1;2*) along with *LeNHX1*, *LeNHX3*, *LeNHX4*, *SIWRKY8, SIWRKY31*, *SIWRKY*39 (*WRKY* gene family) and ERF transcription factors were reported to be highly expressed in a study carried out by Gharsallah et al. (2016) on three tomato genotypes.

The salinity stress can also affect the expression of genes related to nitrogen uptake and transport. In this respect, Abouelsaad et al. (2016) demonstrated a decrease in the expression of mRNA of nitrate transporters *NRT1.1* and *NRT1.2* in both cultivars Manitoba and *S. pennellii*. The same authors observed a higher expression of remarkable affinity ammonium transporters (*AMT1.1* and *AMT1.2*) in Manitoba and a down-regulation of the Gs1 gene (cytosolic glutamine synthetase) in *S. pennellii*.

Other genes whose expression was changed by salt stress are: *SlERF5* gene, part of ERF family gene, which has an important role in the ethylene and abscisic acid signaling pathway (Pan et al., 2012); *SlGSTU23*, *SlGSTU26, SlGSTL3, SlGSTT2, SlDHAR5, SlGSTZ2* involved in primary metabolism, regulation of plant growth and development, anthocyanin’s absorption, detoxification of toxic compounds (xenobiotic, lipid peroxides), etc. (Csiszár et al., 2014); *LeHAK5* gene whose expression was significantly decreased when the Na^+^ concentration was increased (Bacha et al., 2015); *SlARF1*, *SlARF4*, *SlARF8A*, *SlARF19* and *SlARF24* which were upregulated in response to salinity stress (Bouzroud et al., 2018).

The gene *RBCL* (large subunit RUBISCO) whose level of expression was not different as a result of salinity stress, in the presence or absence of ABA synthesis, but whose protein it encodes, showed a significant decrease (Poór et al., 2019).

## Salinity impact on yield and fruit quality

High levels of sodium chloride in soil or in nutritional medium highly affect plant physiological and biochemical processes as well as gene expression, with effects on plant morphology, but also on yield and fruit quality. Most of the research carried out with tomato suggested a positive or no impact of salinity on fruit quality (**Table 6**). Therefore, increases are reported in the lycopene content (De Pascale et al., 2012; Islam et al., 2018; Sellitto et al., 2019), sugar (De Pascale et al., 2012; Islam et al., 2018; Marsic et al., 2018; Botella et al., 2021), total soluble solids (TSS), titratable acidity (TA), organic acids (OA), fruit firmness (Cantore et al., 2012; De Pascale et al., 2012; Martínez et al., 2012; Liu et al., 2014; Zhai et al., 2015; Pengfei et al., 2017; Islam et al., 2018; Rodríguez-Ortega et al., 2019; Maeda et al., 2020; Botella et al., 2021) or cuticle thickness (Agius et al., 2022). According to Agius et al. (2022) a salinity level of up to 5 dS m^−1^ in nutrient solutions may enhance the fruit quality. In a study conducted by Cantore et al. (2012) on two tomato cultivars, salinity increased the content of TSS and had no significant effect on the ascorbic acid content or the TA. Martínez et al. (2012) showed no change in the TSS and TA content at 40 or 80 mM NaCl. At a salinity level of 6.8 dS m^-1^ in soil, the TSS and TA contents in fruits of Buran F_1_ grafted on Maxifort are higher compared to the values determined in fruits grown in soil with the EC of 1.7 dS m^-1^ (Pašalić et al., 2016). Zhang et al. (2016) reported that the salt enrichment in nutrient solution also leads to an increase in the acidity of the tomato fruit. Islam et al. (2018); Costan et al. (2020) and De Pascale et al. (2012) found in their studies that the total soluble solids (Brix index) and citric acid content increased in tomato fruits with salinity increase. In the fruits of tomato cultivar Unicorn the total soluble solids (Brix index) and citric acid content increased by 22% and 20% per dS m^-1^ (Islam et al., 2018). Improvement of fruit quality as a result of salinity was also reported by: Ahmed et al. (2017); Pengfei et al. (2017) in cultivar Pepe; Rodríguez-Ortega et al. (2019) in tomato cultivar Optima; Maeda et al. (2020) in the two tomato cultivars CF Momotaro York and Endeavour. The main factors influencing the fruit quality under salinity stress are harvest day, salinity distribution in the soil or the growth stage (Iglesias et al., 2015; Chen et al., 2016; Zhang et al., 2017). In a study conducted with 4 tomato varieties (Raf, Delizia, Conquista, Tigre) subjected to salinity stress, the content of TSS was significantly decreased when the fruits were harvested 136 days after transplant for cultivar Raf and 90 and 104 days for Delizia; a significant increase of TSS was recorded for Conquista 150 days after transplant and Tigre 136 days (Iglesias et al., 2015). By testing the effect of the uneven vertical distribution of soil salinity on the tomato quality of cultivar Yazhoufenwang, Chen et al. (2016) showed that the content of TSS, OA and vitamin C increased with the soil salt concentration in the upper layer. Zhang et al. (2017), demonstrated that the salinity stress applied from flowering until the fruiting stage improves the TSS content. However, negative effects of high salt levels can be found in the mineral content of tomato fruits. Studies conducted by De Pascale et al. (2012); Hernández-Hernández et al. (2018); Islam et al. (2018); Costan et al. (2020) showed that under salinity stress, the mineral content in tomato fruits (**Table 7**), especially of calcium and potassium, can decrease.

**Table 6:** Salinity impact on yield and citric acid, lycopene, soluble solids contents in tomato fruits.

Tomato cultivar/ hybrid/variety	Salinity level	Citric acid		Lycopene mg/kg f.w.	Soluble solids (°Brix)	Fruit weight (g/fruit)	No. of fruits/plant	Total yield		References
		Unit	Value					Unit	Value	
Tampico F1	0.5 - 4.4 dS-m^-1^	g/kg f.w.	3.2 ↗ 3.4	12.7 ↗ 14.3	4.93 ↗ 5.82	69.1 ↘ 55.5	21.7 ↘ 17.2	tonnes/ha	65.0 ↘ 50.0	De Pascale et al. (2012)
Unicorn	2.5 - 7.5 mS·cm^–1^	%	0.65 ↗ 0.76	115.1 ↗ 137.9	7.66 ↗ 8.01	13.17 ↘ 11.22	–	–	–	Islam et al. (2018)
Belladonna	0, 50 mM	%	2.25 ↗ 3.68	159 ↘ 155	3.85 ↗ 6.60	204.1 ↘ 109.1	22.4 ↗ 26.6	kg/plant	4.54 ↘ 2.9	Costan et al. (2020)
Cerasiforme (Alef)	0- 80 mM	mEq/L	3.90 ↗ 7.75	–	5.63 ↗ 7.78	21.9 ↘ 14.3	13.3 ↘ 12.0	–	–	Martínez et al. (2012)
Tainan ASVEG No. 19	0-150 mM	%	0.62 ↗ 0.93	–	9.2 ↗ 10.7	7.3 ↘ 5.4	–	g/plant	243.9 ↘ 48.8	Liu et al. (2014)
Hualien ASVEG No. 21			0.33 ↗ 1.20	–	7.4 ↗ 13.2	8.7 ↘ 4.6	–		78.7 ↘ 6.9	
Taiwan Seed ASVEG No. 22			0.47 ↗ 0.86	–	8.6 ↗ 12.9	8.1 ↘ 3.6	–		155.5 ↘ 19.3	
Rio Grande	0 - 90 mM	–	–	–	7.74 ↗ 8.87	–	18.89 ↘ 13.00	kg/pot	0.91 ↘ 0.51	Naeem et al. (2020)
Rio Grande	0 - 60 mM	–	–	–	6.13 ↗ 8.24	–	15.44 ↘ 12.33	kg/plant	1.28 ↘ 1.13	Alam et al. (2020)

d.w., dry weight; f.w., fresh weigh.

↑, Increase in parameter value; ↓, decrease in parameter value.

**Table 7:** Salinity impact on mineral content in tomato fruit.

Tomato cultivar/hybrid/variety	Salinity level	Unit	Minerals						References
			N	P	K	Ca	Na	Mg	
Tampico F1	0.5, 2.3, 4.4 dS-m^-1^	% d.w.	2.29 ↘ 1.98	0.275 ↘ 0.233	4.042 ↘ 3.392	0.319 ↘ 0.288	–	–	De Pascale et al. (2012)
Unicorn	2.5, 5, 7.5 mS cm^–1^	% d.w.	–	0.637 ↘ 0.287	2.44 ↘ 2.10	0.127 ↘ 0.087	–	0.13 ↗ 0.17	Islam et al. (2018)
Belladonna	0, 50 mmol/L	g/kg d.w.	17.61 ↗ 17.78	1.38 ↗ 1.58	24.05 ↘ 21.72	0.82 ↘ 0.55	0.62 ↗ 1.31	0.59 ↗ 0.62	Costan et al. (2020)
Durinta F1	7, 21, 37, 49, 64 mM	% d.w.	2.22 ↘ 2.02	–	3.91 ↘ 3.55	0.13 ↗ 0.15	0.08 ↗ 0.26	0.15 ↘ 0.12	Giuffrida et al. (2009)
Huno F1	0, 100 mM	g/kg d.w.	23.14 ↘ 21.78	–	9.16 ↘ 8.31	6.34 ↘ 2.92	0.98 ↗ 4.30	2.31 ↗ 2.34	Hernández-Hernández et al. (2018)

d.w.,– dry weight.

↑, Increase in parameter value; ↓, decrease in parameter value.

Regarding tomato yield under saline stress, the Division of Agriculture and Natural Resources of University of California specifies that a soil salinity of 7.6 dS m^-1^ may reduce both tomato plant emergence and crop yield by 50% (Division of Agriculture and Natural Resources, 2022), but these effects are closely related to the tomato cultivar. The study performed by De Pascale et al. (2012) showed that at 4.4 dS m^-1^ the mean fruit weight, the number of fruits per plant and the total yield of tomato decreased compared to the control (0.5 dS m^-1^) by 19.68%, 20.74%, and 23.07%, respectively. According to Islam et al. (2018) an increase in soil salinity from 2.5 at 7.5 dS m^-1^ causes a 14.81% reduction in the mean fruit weight of the cultivar Unicorn. In addition, Liu et al. (2014) reported that the yield of three cherry tomato cultivars grown inpeat moss, perlite and sand mix (2:1:1) was affected differently by the same levels of salinity. At 150 mM NaCl the mean fruit weight of Tainan ASVEG No. 19, Hualien ASVEG No. 21 and Taiwan Seed ASVEG No. 22 was reduced by 26.03%, 47.13%, and 55.56% respectively, compared to the control, and the total yield decreased from 243.9, 78.7 and 155.5 g/plant to 48.8, 6.9, and 19.3 g/plant, respectively. Costan et al. (2020) reported that, although the number of fruits per plant increased with the salinity rises in the hydroponic system (from 0 at 50 mM), the yield of the tomato cultivar Belladonna was reduced by more than 36%. Noshadi et al. (2013) found the highest yield (47.15 t·ha^-1^) was recorded when the irrigation water EC was of 2 dS m^-1^. At 0.6 dS m^-1^, 38.02 t·ha^-1^ were harvested and at 4 dS m^-1^ about 31.57 t·ha^-1^, whereas the lowest yield was at 8 dS m^-1^ EC (21.20 t·ha^-1^). Therefore, according to the results of the latter study, a slightly saline soil or hydroponic cultivation can enhance tomato yield.

## Recommendations for alleviating the effects of salinity on tomato

The negative effects of salinity on tomato plants can be alleviated by using different strategies like plant priming or genetic modification.

Plant priming represents a promising method to reduce the time required for a plant exposed to abiotic stress to respond efficiently to the stressor and, thereby, to increase the tolerance to stress conditions (Aranega-Bou et al., 2014). Effective priming agents against salt stress in tomato, which have been studied over years are elements (Fe, Si, K, N), plant growth regulators (ACC, IAA, SA, melatonin), reactive species (S-nitrosoglutathione, sodium hydrosulfide, sodium nitroprusside), vitamins (ascorbic acid - AsA), aminoacids, natural extracts (seaweed), polymers (chitosan), osmoprotectants (glycine betaine, proline), polyamines (spermidine) or plant growth promoting microorganisms (bacteria, fungi or arbuscular mycorrhizal fungi) (Choudhary et al., 2022; Gedeon et al., 2022; Zulfiqar et al., 2022). The results showed in most of the cases an enhancement of the tolerance of plants to various concentrations of salt, by decreasing the osmotic stress, enhancing the activity of the antioxidant system, increasing the growth and yield or by improving the fruit quality. For instance, the application of Fe increased the ascorbic acid content in the fruits of tomato along with the increment in salinity level; the Si addition stimulated an early accumulation of TSS in the fruits of tomatoes, but did not influence the quality of the taste; in another study, the presence of Si decreased the SOD activity, suggesting a reduction in ROS production; also, the treatment with Si increased the β-carotene and vitamin C content; the addition of 5 mM K^+^ regulated the ascorbate–glutathione cycle, the activity of antioxidant enzymes, the carbohydrate metabolism and increased the proline content; nitrogen applied at different concentrations (25, 75, 150 kg N ha^−1^) had a positive impact on the proline content and on the activity of P5CS enzyme, also affected the activity of various enzymes: proline dehydrogenase, nitrate reductase, nitrite reductase, glutamine synthetase and glutamate synthase, glutamate dehydrogenase under NaCl stress (Tantawy et al., 2013; Iglesias et al., 2015; Muneer and Jeong, 2015; Singh et al., 2016; Costan et al., 2020; Khan et al., 2021). The application of plant growth regulators such as ACC decreased the osmotic stress in ‘Ailsa Craig’ tomato cultivar; spraying the tomato plants with IAA (100 and 200 ppm) increased the TSS content of fruit juice and the chlorophyll content of the leaves; the exogenous application of salicylic acid decreased the ethylene synthesis and increased the polyamine endogenous concentration; in another study, salicylic acid applied foliar increased the TSS and the vitamin C content; the treatment of the seeds with salicylic acid (1 mM) and H_2_O_2_ (50 mM) increased the TSS, proteins, POD, CAT, SOD and MDA content; the treatment with 20 and 50 µM melatonin improved the activity of the antioxidant system, the proline and carbohydrate metabolism, also the ascorbate/reduced glutathione cycle in ‘Five Start’ tomato cultivar; in another studies, melatonin improved the root architecture, reduced the production of reactive oxygen species, enhanced the activity of enzymatic antioxidants and the photosynthesis (Gharbi et al., 2016; Gaba et al., 2018; Siddiqui et al., 2019; Alam et al., 2020; Altaf et al., 2020, 2021; 2022b; Borbély et al., 2020; Naeem et al., 2020; Hu et al., 2021; Ali et al., 2021b). The application of S-nitrosoglutathione and NaHS promoted the accumulation of NO and H_2_S, alleviating the deleterious effects of oxidative stress; the use of sodium nitroprusside increased the content of non-enzymatic and enzymatic antioxidants, up-regulated the NO level in leaves, enhanced the activity of Calvin cycle, overcame the stomatal limitations and protected the photosystem II from damages (da-Silva et al., 2018; Taheri et al., 2020; Li et al., 2022). Alves et al. (2021) by soaking the tomato ‘Micro-Tom’ seeds for one hour in 100 mM AsA, observed that the tolerance of plants to salt stress was enhanced by modulating the antioxidant mechanisms. The content of CAT, APX, POX, GPX, GR, GSH, SOD, chlorophyll, and carotenoids in the leaves of primed plants was higher than in the control. Chen et al. (2021) by spraying 0.5 mmol/L AsA solution on the leaves of cv. ‘Ligeer87-5’ exposed at 100 mmol/L NaCl reported an attenuation of the photoinhibition and oxidative stress damage in chloroplasts, dissipation of excitation energy in PSII antennae, stimulation of chlorophyll synthesis and reduction of damaging effects on photosynthesis in tomato leaves. The foliar application of an aminoacid (Botamisol as free L-amino acids) at different concentrations (0, 2, 4 g·L^-1^) increased the proline level in the leaves of tomato plants exposed to salinity (8 and 10 dS·m^-1^) (Jannesari et al., 2016). The application of a seaweed extract (100 mL of *P. gymnospora* 0.2% w/v) improved the growth, yield and quality of ‘Rio Fuego’ tomato cultivar (Hernández-Herrera et al., 2022). The use of chitosan solution at different concentrations (0.03% and 0.05% or 50, 100 and 150 mg/L) for spraying the tomato leaves, enhanced the salt tolerance of tomato at 100 mM NaCl applied as a root drench, promoted the growth and development of plants and increased the chlorophyll contents (Ullah et al., 2020; Özkurt and Bektaş, 2022). The exogenous application of spermidine (Spd) on tomato cv. ‘Ailsa Craig’ seedlings grown under salt stress resulted in higher photosynthesis and biomass, better ionic and osmotic homeostasis, and enhanced ROS scavenging capacity (Raziq et al., 2022). Siddiqui et al. (2017) found that the chlorophyll *a* and *b* contents, proline, activity of CAT, SOD, POD, GR and APX were increased and H_2_O_2_ and MDA production in tomato var. Five Star was reduced as a result of exogenous spermidine application on seedlings. The foliar application of 10 and 20 mg/L proline during the flowering stage of cultivars ‘Rio Grande’ and ‘Heinz-227’ led to an increase in the dry mass of leaves, stems and roots, improved various chlorophyll fluorescence parameters, increased the potassium and phosphorous content and reduced the accumulation of Na^+^ in different organs, compared with control (Kahlaoui et al., 2014). The effects of the exogenous application of glycine betaine (GB) on different tomato cultivars have been assessed in a few studies and both positive and negative correlations were found between GB exogenous application and salt tolerance in tomato. Chen et al. (2009) found that the exogenous use of 5 mM GB in half-strength Hoagland could alleviate the salt stress effects in tomato cv. ‘F144’ and cv. ‘Patio’ through changing the expression abundance of some proteins. Sajyan et al. (2019) irrigated the tomato ‘Sila’ plants with saline water (with EC between 2 and 10 dS m^-1^) and exogenous GB in various doses (4.5, 6 and 7.5 g/L) and observed a positive effects on leaf number, stem diameter, number of flowers, number of fruits, no evident effects on the number of clusters, fruit set, the weight of individual fruit, yield and fruit diameter were observed and a reduction in the fruit ripening process at 7.5 g/L GB.

Plant growth-promoting rhizosphere bacteria (PGPB) can alleviate the effects induced by salt stress by production of phytohormone (e.g. auxin, cytokinin, and abscisic acid), ACC-deaminase, ammonia, IAA, extracellular polymeric substance (EPS), induction of synthesis of plant osmolytes and antioxidant activity, increasing the essential nutrient uptake or/and by reducing ethylene production (Kumar et al., 2020). *Sphingobacterium* BHU-AV3 (Vaishnav et al., 2020), *Bacillus megaterium* strain A12 (Akram et al., 2019), *Enterobacter* 64S1 and *Pseudomonas* 42P4 (Pérez-Rodriguez et al., 2022), *Bacillus aryabhattai* H19-1 and *Bacillus mesonae* H20-5 (Yoo et al., 2019) are some of the PGPB that have been proved to increase tomato tolerance to salt stress. For example, inoculation of tomato cv. ‘Kashi amrit’ plants with *Sphingobacterium* BHU-AV3 exhibited a less senescence in plants exposed to 200 mM NaCl, being determined that the proline content was increased, ion balance was maintained and the ROS was lower compared to the non-inoculated plants. In BHU-AV3-inoculated plant leaves superoxide content, cell death and lipid peroxidation were significantly reduced (Vaishnav et al., 2020). *Enterobacter* 64S1 and *Pseudomonas* 42P4 under salt stress reduced electrolyte leakage and lipid peroxidation and increased chlorophyll quantum efficiency (Fv/Fm), proline and antioxidant nonenzymatic compounds (carotenes and total phenolic compounds) contents in tomato leaves (Pérez-Rodriguez et al., 2022). A combination of arbuscular mycorrhizal fungi (*Claroideoglomus etunicatum*, *Funneliformis mosseae*, *Glomus aggregatum*, *Rhizophagus intraradices*), bacteria and fungi (*Trichoderma*, *Streptomyces*, *Bacillus*, *Pseudomonas*) improved the tomato fruit quality and the antioxidant content of ‘Pixel F1’ tomato cultivar exposed to soils electrical conductivity of 1.5, 3.0, 4.5, and 6.0 (Sellitto et al., 2019).

Some researchers have focused not only on assessing the individual effects of a potential priming agent against salt stress in tomato plants, but also their combined effect. For example, Attia et al. (2021) studied the effects of foliar application of chitosan dissolved in acetic acid (Ch ACE), ascorbic acid (Ch ASC), citric acid (Ch CIT) and malic acid chitosan (Ch MAL) on tomato cultivar 023 irrigated with saline water (100 mM NaCl). These treatments alleviated the negative effects of salinity on tomato plants by increasing the photosynthetic pigments, osmoprotective compounds, and potassium content and lowering MDA, H_2_O_2_ and Na^+^ levels in leaves. Chanratana et al. (2019) used as a bioinoculant chitosan-immobilized aggregated *Methylobacterium oryzae* CBMB20 to improve the salt tolerance of cv. ‘Yeoreum Mujeok Heukchima’ and the results showed that plant dry weight, nutrient uptake, photosynthetic efficiency, and the accumulation of proline have been enhanced. Furthermore, the oxidative stress exerted by salt stress was alleviated and the electrolyte leakage and the excess Na^+^ influx into the plant cell were reduced.

Tomato genetic modification techniques have already proven their efficiency and accuracy in protecting plants against salinity stress by improving their genome. Gene transformation, gene editing, quantitative trait loci (QTLs) analysis, gene-pyramiding, and genetic engineering (overexpression) are some examples of molecular genetic tools that have helped in the development of salt-tolerant tomato plants.

Gene transformation has mainly focused on transferring genes of various origins, which can be good candidates to increase the tolerance to salinity stress, into tomato plants. Salt tolerant tomato plants were successfully obtained by Gilbert et al. by transferring the gene HAL1 from *Saccharomyces cerevisiae*, involved in Na^+^ transport and K^+^ regulation, which improved the *in vivo* and *in vitro* salt tolerance of transgenic tomato plants, by promoting the retention of K^+^ and the growth of the plants (Gisbert et al., 2000); by Goel et al., who demonstrated that by transforming the tomato cultivar ‘Pusa Ruby’ with the bacterial *codA* gene from *Arthrobacter globiformis* encoding for choline oxidase, the production of glycine betaine was induced, the content of relative water, chlorophyll and proline increased, also the overall tolerance of the plants under saline stress was improved (Goel et al., 2011); by Jia et al., who transferred the *BADH* gene from *Atriplex hortensis* in ‘Bailichun’ tomato cultivar, obtaining a normal growth and development of the plants treated with 120 mM NaCl (Jia et al., 2002); by Li et al., who isolated the *SpPKE1* a lysine-, glutamic- and proline-rich type gene from the abiotic resistant *Solanum pennellii* LA0716 and transferred it to *S. lycopersicum* cv. M82 or by transferring the *Osmotin* gene from tobacco into tomato plants, an increased tolerance to salt stress was obtained, highlighted by better cell signaling, ROS scavenging, the content of carbohydrates, amino acids, polyols and performance of the antioxidant and photosynthetic systems (Goel et al., 2011; Li et al., 2019a; Rao et al., 2020).

The only genetic editing technique that has been reported to be used in improving the tomato tolerance to salinity is clustered regularly interspaced short palindromic repeat (CRISPR)-Cas9 (CRISPR-associated nuclease 9) a modern, easy and very effective genome editing tool (Salava et al., 2021; Altaf et al., 2022a). However, the researches on increasing tomato tolerance to salt stress by using CRISPR/Cas9 are still limited. So far, this tool was used to precisely edit the hybrid proline-rich proteins domain (*HyPRP1*) involved in different biotic and abiotic responses. The deletion of the *SlHyPRP1* negative-response domain led to salt tolerance as high as 150 mM NaCl, improving the germination and the growth of the plants (Tran et al., 2021). The same results were obtained earlier by Li et al., who also observed that by silencing the negative regulator *HyPRP1* the expression of the genes responsible for the production of SOD and CAT was enhanced (Li et al., 2016). In addition, CRISPR/Cas9 technology was used to knock out the *SlABIG1* gene in tomato exposed to salinity, resulting plants with improved chlorophyll and proline content, photosynthetic system, root dry weight and decreased concentrations of ROS, MDA and Na^+^ (Ding et al., 2022). By using the same tool, Wang et al., demonstrated the importance of the plasma membrane Na^+^ /H^+^ antiporter *SlSOS1* in the salt tolerance of tomato, by creating two mutant alleles (*Slsos1-1* and *Slsos1-2*) which showed a significant increase in the Na^+^/K^+^ ratio and the salt sensitivity, as compared with the wild type (Wang et al., 2021). Bouzroud et al., by generating tomato *SlARF4*-crispr (*arf4-cr*) plants showed the importance of *Auxin Response Factor* 4 (ARF4) in the tolerance of tomato plants to salinity (Bouzroud et al., 2020). Regarding the other two known genetic editing techniques (zinc finger nucleases - ZFNs and Transcription Activator-Like Effector Nucleases - TALENs) no reports are available on tomato tolerance (Salava et al., 2021; Altaf et al., 2022a).

Due to the QTLs mapping, different loci related to the oxidative defence system, Na^+^/K^+^ homeostasis, or developmental stages were identified in playing an important role in increasing the tomato tolerance to salinity. Therefore, Frary et al., identified 125 QTLs for antioxidant compounds under saline and non-saline conditions in *S. pennellii* tomato introgression lines, and their parental lines, salt-resistant wild tomato (*S. pennellii* LA716) and the salt sensitive cultivated *S. lycopersicum* Mill. cv. M82 that could be beneficial in developing salt-tolerant cultivars. Under the salt stress (150 mM NaCl), the wild tomato and different introgression lines accumulated more antioxidant compounds (phenolics, flavonoids, SOD, CAT, APX) than the cultivated tomato (Frary et al., 2010). The same wild tomato ascension, the wild *S. lycopersicoides* LA2951 and two introgression lines derived from them were used to identify QTLs for tolerance to salinity in the seedling stage by Li et al. Four major QTLs were detected on chromosomes 6, 7 and 11 in *S. pennellii* IL library, while in *S. lycopersicoides* IL library, six major QTLs were found on chromosomes 4, 6, 9 and 12. The authors concluded the possibility to create hybrids with QTLs coming from these two ascensions (Li et al., 2011). Foolad et al., detected and validated a number of five QTLs for tomato salt tolerance during vegetative growth in a population (BC_1_) resulted from the crosses between the breeding line NC84173 (*Lycopersicon esculentum* Mill.) and *L. pimpinellifolium* (Jusl.) Mill. accession LA722. One minor QTLs was identified on chromosome 3 in the interval CT82–TG515, two major QTLs on chromosomes 1 and 5, and the other two on chromosomes 6 and 11 (Foolad et al., 2001). Villalta el al., found QTLs for salt tolerance during reproductive stage in two populations of F7 tomato lines (P and C) resulted from ‘cerasiforme’ variety (salt sensitive genotype), as female parent, and two lines tolerant to salt tolerant, as male parents: *S. pimpinellifolium*, the P population (142 lines), and *S. cheesmaniae*, the C population (116 lines). The authors suggested that the QTLs detected by them can be used to increase the fruit yield of tomato plants under salt stress, being good candidates for increasing the tomato tolerance to salinity. The QTLs for fruit yield were detected in chromosome 5, the specific loci being *fn5.2* and *tw8.1* found in C population and *fn10.1* which overlaps *tw10.1* and *fw8.1* loci in P population. Under saline conditions the fruits set percentage per truss, fruit number per plant and the total fruit weight per plant increased (Villalta et al., 2007). Other candidates for QTL can be those associated with Na^+^/K^+^ homeostasis are the genes encoding HKT1-like transporters (*SlHKT1;1* and *SlHKT1;2*), with tonoplast NHX Na^+^/H^+^-antiporters (*SlNHX3* and *SlNHX4*), with the content of α-tocopherol in tomato fruits (chromosomes 6 and 9), or with tocopherol biosynthesis (chromosomes 7, 8, and 9) (Egea et al., 2022).

Gene pyramiding, which consists in combining multiple traits in a single genotype, represents another method that can help to obtain tomato plants tolerant to salinity stress, but the researches are still limited. Some strategies that have been proposed refer to pyramiding the ascorbic acid (AsA) biosynthetic pathway, the ascorbate–glutathione pathway, or different QTLs. For improving the AsA content in tomato, Li et al. pyramided the biosynthetic genes involved in the D-Man/L-Gal pathway of ascorbate, resulting the pyramiding lines GDP-Mannose 3′,5′-epimerase (*GME*) × GDP-d-mannose pyrophosphorylase (*GMP*), GDP-l-Gal phosphorylase (*GGP*) × l-Gal-1-P phosphatase (*GPP*) and *GME* × *GMP* × *GGP* × *GPP*. The results showed increased concentrations of total ascorbate in leaves and fruits and improved AsA transport capacity, light response and salinity stress tolerance. In addition, the fruit weight (significantly decreased in *GGP × GPP* lines), fruit size (significantly decreased in *GMP × GME* and *GGP × GPP* lines), and soluble solid (significantly increased in *GMP × GME* and *GMP × GME × GGP × GPP* lines) were affected by pyramiding maybe because of the influence of different primary metabolism pathways (sugar, acid, and cell wall metabolism) as stated by the authors (Li et al., 2019b). By pyramiding the genes of ascorbate-glutathione pathway, isolated from *Pennisetum glaucoma (Pg)* (*PgSOD, PgAPX, PgGR, PgDHAR* and *PgMDHAR*) Raja et al., obtained tomato lines with better germination rate, survival rate, photosynthetic and antioxidant activity, reduced ROS production, and membrane disruption, under 200 mM NaCl (Raja et al., 2022). Pyramiding QTLs can be an effective method to improve the tomato salt tolerance. The pyramiding of QTLs takes place by using a marker assisted selection (MAS). Some authors proposed the use of different QTLs associated with salt tolerance during seed germination or vegetative growth in tomato (Foolad, 2004).

Another way to enhance the tomato salt tolerance is to overexpress specific genes that can increase the tomato tolerance to salt stress. Some authors highlighted the importance of various genes in the salt stress in transgenic plants and, in this respect, Hu et al. (2014) demonstrated that the overexpression of *LeERF1* and *LeERF2* genes have a positive impact on tomato plants exposed to salinity stress. Good results regarding different physiological and biochemical parameters (i.e. root length, chlorophyll, proline and antioxidant enzymes contents) were obtained in the transgenic tomato, where the expression of other genes related to salinity stress was up-regulated (*RBOHC, TAS14*, *HVA22*, *PR5* and *LHA1*). The overexpression of *SlERF5* gene (ethylene response factor) in transgenic tomato led to an increased tolerance to salinity by improving the relative water content (Rao et al., 2020). Albacete et al. (2014) recorded improved fruit yield, hormone concentrations, and sugar content in transgenic tomato due to the overexpression of a gene coding for isopentenyl transferase, an enzyme involved in cytokines biosynthesis – *IPT* gene and a cell wall invertase gene – *CIN1*. Cai et al., 2016 showed the importance of *SlDof22* gene, coding for Dof proteins responsible for abiotic stress response, gibberellins regulation, and evolution of cell cycle, in improving the tomato tolerance to salinity stress. Other genes whose expression increased the tomato plant biomass production and yield under salinity stress were *CDF3*, which regulated important genes for redox homeostasis, photosynthesis process or primary metabolism (Renau-Morata et al., 2017). NAC transcription factor SlTAF1 is another gene described as a good candidate for increasing the salinity tolerance of tomato and other species. It’s silencing in transgenic plants increased the damages related to salinity (Devkar et al., 2020).

## Conclusions and future perspectives

Soil salinity represents one of the main causes of agricultural yield losses worldwide. Natural factors such as topography, and type of geological material, but especially anthropogenic activities like inappropriate agricultural practices (i.e. excessive fertilization, irrigation without proper drainage, and leaching) intensify the soil salinization process. Plants are directly impacted by the increases in soil salt concentration through reduced water and nutrient uptake by roots. In tomato plants, salinity stress affects positively ornegatively the germination process, the morphological traits, the physiological features, the biochemical and molecular parameters, and also the yield. Usually, the germination, morphology and physiology of tomato plants are negatively influenced by the saline stress. When the soil salinity increases, its water potential drops to a point close to the root water potential, slowing down the process of water uptake by roots, thus causing drought stress-related symptoms. Also, in saline soils, nutrients in the form of cations (Mg^+^, Ca^+^, K^+^, ${\text{NH}}_{4}^{+}$ ) and anions ( ${\text{NO}}_{3}^{-}$ , ${\text{PO}}_{4}^{3-}$ ) compete with Na^+^ and Cl^−^ to be transported inside the plant. Na^+^ competes with ${\text{NH}}_{4}^{+}$ and K^+^ cations decreasing their absorption, while Cl^−^ competes with ${\text{NO}}_{3}^{-}$ anions decreasing its uptake. Therefore, along with a deficiency in the nutrient uptake, ion toxicity takes place due to excessive concentrations of Na^+^ and Cl^−^, consequently affecting plant growth and development. Regarding the effects on gene expression, the salinity stress can down-regulate or up-regulate the expression of genes in tomato plants. A similar situation also occurs with regard to the biochemical parameters which can either be enhanced by the saline stress or can be decreased. Generally, most of the increases and the decreases recorded for the biochemical parameters and the up- or down-regulation of genes represent adaptive responses to stress by plants that try to improve their homeostasis and resistance. However, the decreases can also be the result of biochemical pathways dysregulations. The quality of tomato fruit benefits from saline conditions in most cases, maybe due to lower water content and accumulation of biomolecules such as sugars, amino acids, and inorganic solutes that contribute to osmotic adjustments.

The results of the studies carried out over the last 10 years have shed more light on the impact that saline stress can have on tomato plants. However, for a clearer image of the effects of salinity on tomato plants, more studies should be carried out in the field, in salt-affected soils, taking into account the individual and cumulative interactions of the factors involved.

The deleterious effects of salinity on tomato plants can be alleviated by using different strategies like plant priming or genetic modification techniques. The results are very promising, but at this moment, they are relatively limited and at their beginnings. In addition, most of the research has focused on developing salt-resistant tomato plants and testing them for the needed characters, but to develop commercial lines, research carried out in saline fields are needed.

Considering the FAO predictions that by 2050 more than 50% of arable land will become saline, urgent measures should also be taken to reduce the salinization process such as better water drainage and leaching of salts; a decrease in the quantity and number of fertilizers applied and water used in irrigation; proper crop selection or reduction of the degree of tillage systems. Therefore, researchers should focus more their attention on methods to desalinate the soils, on studies regarding the development of fertigation schemes that promote a better management of water and fertilizers applied according to the plant requirements, on the production of new varieties resistant to salinity, or in improving the existing species.

## Author contributions

VS, GM and MR: Conceptualization. MR and GM: Formal analysis. MR and GM: Investigation. MR and GM: Writing—original draft preparation. VS: Supervision and validation. VS, GM and MR: Writing - review & editing. MR and GM contributed equally to this work and share first authorship. All authors have read and agreed to this version of the manuscript. All authors contributed to the article and approved the submitted version.
